# Automated behavioural analysis reveals the basic behavioural repertoire of the urochordate ***Ciona intestinalis***

**DOI:** 10.1038/s41598-019-38791-5

**Published:** 2019-02-20

**Authors:** Jerneja Rudolf, Daniel Dondorp, Louise Canon, Sonia Tieo, Marios Chatzigeorgiou

**Affiliations:** 1 Sars International Centre for Marine Molecular Biology, University of Bergen, Thormøhlensgate 55, 5006 Bergen, Norway; 2 École Nationale Supérieure de Chimie de Montpellier, 240 Avenue du Professeur Emile Jeanbrau, 34090 Montpellier, France; 3 University Paris Diderot-Paris7, 5 rue Thomas Mann, 75013 Paris, France

## Abstract

Quantitative analysis of animal behaviour in model organisms is becoming an increasingly essential approach for tackling the great challenge of understanding how activity in the brain gives rise to behaviour. Here we used automated image-based tracking to extract behavioural features from an organism of great importance in understanding the evolution of chordates, the free-swimming larval form of the tunicate *Ciona intestinalis*, which has a compact and fully mapped nervous system composed of only 231 neurons. We analysed hundreds of videos of larvae and we extracted basic geometric and physical descriptors of larval behaviour. Importantly, we used machine learning methods to create an objective ontology of behaviours for *C*. *intestinalis* larvae. We identified eleven behavioural modes using agglomerative clustering. Using our pipeline for quantitative behavioural analysis, we demonstrate that *C*. *intestinalis* larvae exhibit sensory arousal and thigmotaxis. Notably, the anxiotropic drug modafinil modulates thigmotactic behaviour. Furthermore, we tested the robustness of the larval behavioural repertoire by comparing different rearing conditions, ages and group sizes. This study shows that *C*. *intestinalis* larval behaviour can be broken down to a set of stereotyped behaviours that are used to different extents in a context-dependent manner.

## Introduction

Close observation of living animals can reveal the large repertoire of behaviours they use to interact with the world. Animals can crawl, swim, run and fly to move from one place to another. The availability of modern computational analysis tools and accessible hardware for recording videos with high temporal resolution have made it possible to observe and quantify behaviour in a more comprehensive, accurate and automated approach^1–4^.

**Figure 1:**
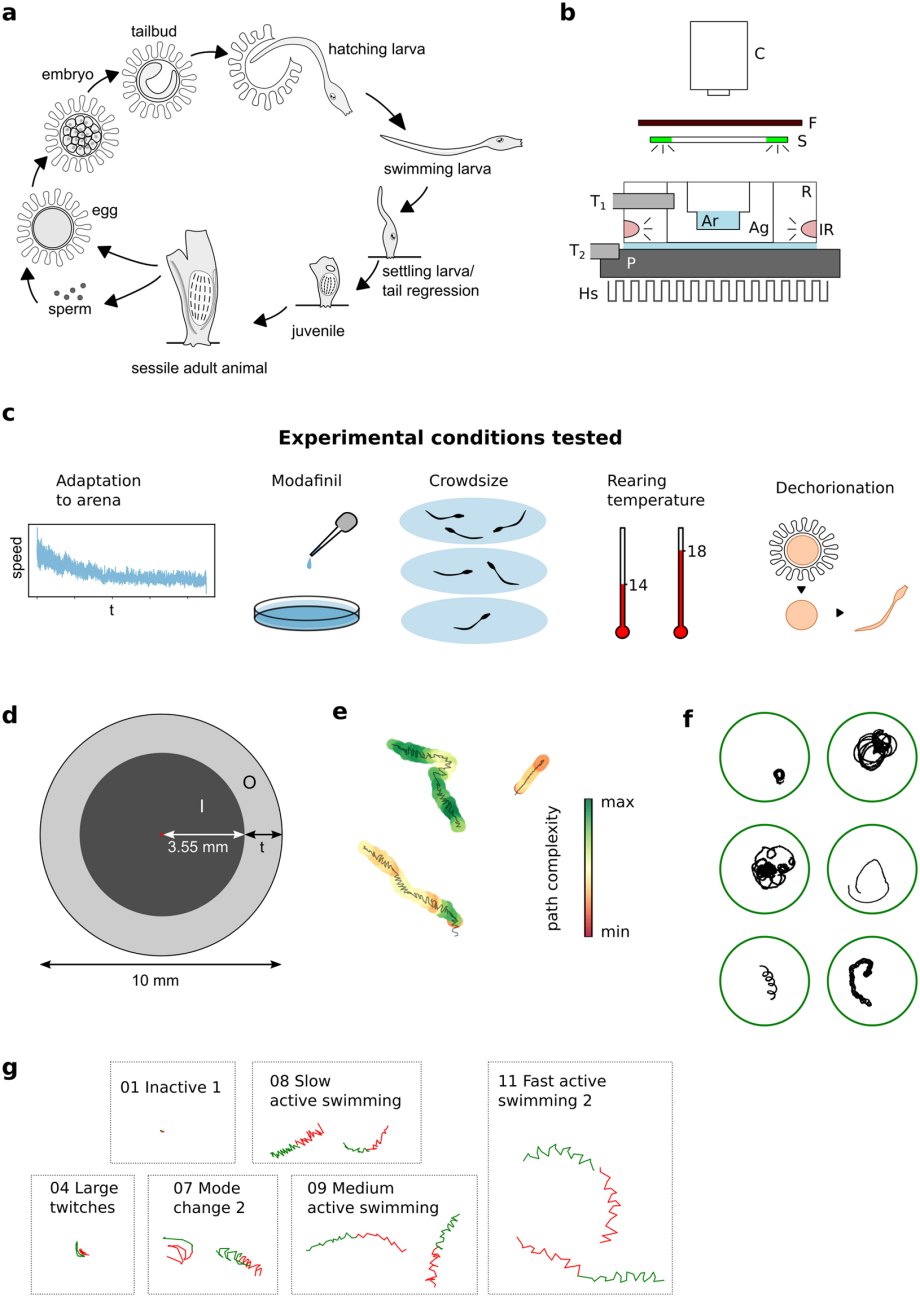
Setup, experimental design and analysis methods. (**a**) Life cycle of *Ciona intestinalis*. In our lab the onset of hatching from the chorion takes place at 25 and 36 hours post fertilization (h.p.f) at 18 °C and 14 °C respectively. The onset of the light-off response in swimming larvae takes place at ca 28.5 and 43 h.p.f at 18 °C and 14 °C respectively. The onset of tail regression takes place at approximately 35 and 52 h.p.f. at 18 °C and 14 °C respectively. (**b**) Setup: C- camera, F- IR filter, S – ring with stimulation LED-s, T1 – Thermometer 1 measuring local temperature in the agarose, T2 – Thermometer 2 measuring cooling plate temperature, P – cooling plate, Hs – heat sink, R – PLA ring holding the arena, Ag– agarose, Ar– Arena with animals, IR – IR illumination. (**c**) Schematic representation of the experimental condition tested for effects on the behavioural repertoire. (**d**) Arena dimensions and areas used in thigmotaxis measures I- inner arena with radius 3.55 mm and O– outer thigmotaxis zone; with t = 1.45 mm the zones have equal surface area and the width of the thigmotaxis zone is above one animal body-length. (**e**) Examples of different local complexity of a trace. Each trajectory is coloured by local complexity which is calculated over a 3 s window and the total span of each trace is 6 s. (**f**) Some example trajectories of wild type animals swimming for 5 min. (**g**) Examples of traces spanning 50 frames based on which the current behavioural mode was calculated. In green are the 25 frames before the current time-point and in red the 25 frames later.

Researchers have used automated behavioural analysis to divide and classify behaviour into distinct modules in several laboratory organisms, including worms, flies, zebrafish and mice. Recent analysis methods that allow for the recognition and segmentation of morphologically and behaviourally diverse animals, offer the opportunity to perform quantitative behavioural analysis of phylogenetically key organisms across different taxa^5,6^. One such organism with great potential for quantitative behavioural analysis combined to evolutionary and neurobiological studies is the larval form of the tunicate *Ciona intestinalis* (Fig. 1a).

Tunicates are the closest relatives of vertebrates and have been successfully opted as models to study the evolution of chordates^7^. They have larvae with a chordate body plan and development^8^. The two most intensively studied tunicate species are *Ciona intestinalis* and *Ciona robusta*. *Ciona* features a defined cell lineage, an extensive genetic toolkit and a sequenced genome that shares a high number of homologous genes to its vertebrate counterparts^9^. *Ciona* is particularly useful for investigating the chordate origins of many biological processes and has been very successful as a model for studying the development and evolution of the chordate nervous system^10^. It possesses a dorsal central nervous system for which a documented synaptic connectome of the 177 CNS neurons is now available^11,12^. Together with a publication of the peripheral nervous system connectome demonstrating the presence of an additional 54 neurons^13^, these studies make *C*. *intestinalis* the second organism with a complete connectome available after *Caenorhabditis elegans*^14^.

An adult *C*. *intestinalis* animal can release hundreds of eggs together with sperm. Gametes undergo fertilization and through a series of stereotyped developmental steps, the embryos take the form of hatching lecithotrophic larvae. When the larvae hatch from the chorion, they find themselves in the water column. Following hatching, larvae swim upwards towards the water surface by negative gravitotaxis using the otolith cell. Ablation experiments have shown that animals lacking the ocellus are also capable of this behaviour, indicating that the ocellus is not involved in gravitotaxis^15,16^. Later on, larvae exhibit negative phototaxis, swimming away from the bright surface to deeper waters in a behaviour that possibly aims to identify suitable substrates for settlement^17,18^. According to a previous report, the swimming larvae display three types of swimming activity: tail flicks, spontaneous swimming and shadow response^19^. Larvae under constant illumination swim more frequently and for periods that are more extensive earlier in life, up to 2 hours post hatching. A behaviour that develops later in development is the shadow response, where dimming of light results in symmetrical swimming. After two hours post hatching the tail beating frequency increases^19^. Notably, *Ciona* larvae exhibit both sensitization and habituation to light^20,21^. A recent study reported that distinct groups of photoreceptors mediate negative phototaxis and dimming behaviour in *Ciona* larvae^22^. The authors of this study obtained this functional insight through behavioural analysis on mutant lines, demonstrating the power of genetics in combination with behavioural analysis and knowledge of the larval connectome. However, this is not the first attempt to link behaviour to its genetic underpinnings in *Ciona* as it has been shown that targeted knockdown of opsin1 results in a loss of the light OFF response in larvae^23^. Beyond phototactic and gravitotactic behaviours there is evidence hinting to the possibility that the larvae can exhibit chemotactic^24^ and mechanosensory behaviours^25,26^. However, these have remained largely unexplored.

In the present study, we expand on the existing work by providing a detailed characterization of the behavioural output of the *C*. *intestinalis* larvae to complement the growing insights into the structure and function of this prototype chordate nervous system. To achieve this, we collected a large dataset of recordings of free-swimming *C*. *intestinalis* larvae on a custom IR-illuminated and temperature-controlled set-up (Fig. 1b). We analysed the recordings using the automated tracking software ToxTrac^27^ and custom written Python scripts to obtain positional data and descriptors like speed and its variability, turning angles, and path complexity (Fig. 1c,e). To describe behaviour both accurately and objectively, we used unsupervised clustering methods to identify distinct behavioural clusters in our dataset, ultimately arriving at a quantifiable distribution of detectable behavioural components. With our experimental approach, we were able to reveal that *C*. *intestinalis* larvae can enter a state of sensory arousal and that they can exhibit a strong thigmotactic behaviour. Interestingly, the anxiotropic drug modafinil is able to modulate thigmotaxis. Finally, we demonstrate that our setup and analysis approach is sensitive enough to study how behavioural descriptors can change under the influence of different rearing conditions, post-embryonic development and in response to different sensory cues (Fig. 1c).

## Methods

### Animals and rearing conditions

We collected adult *Ciona intestinalis* from the following sites in the Bergen area: Døsjevika (Bildøy Marina AS), Døsjevegen, 5353, Straume and Telavåg. We kept the animals in a purpose-made facility, using filtered seawater at 10 °C with a pH of 8.2 under constant illumination to stimulate egg production. We obtained eggs and sperm from individual animals to perform *in vitro* fertilisation. To study the effects of dechorionation on behaviour we selected a subset of the eggs for dechorionation using Na-Thioglycolate and mechanical dechorionation^28^. Both eggs with and without chorion were fertilized at the same time and incubated in artificial sea water (ASW, Red Sea Salt) at either 14 or 18 °C. The ASW pH was 8.4 at 14 °C and 8.25 at 18 °C. The salinity of the ASW was 3,3–3,4% and 3,5% in the facility tanks where adult animals were housed. We reported the post hatching age of animals with reference to the onset of hatching of larvae from the chorion.

### Set-up for behavioural experiments

We built our behavioural setup (See Fig. 1b for schematic) around a Nikon SMZ1500 stereomicroscope fitted with a HR Plan Apo 1x (N/A 0.131/WD54 mm). To print the custom-made parts of the setup we used a Weistek WT280A 3D-printer. Using a 3D-printed PLA mould, we made single-use agarose arenas (0.8% in ASW, by Invitrogen, USA,). The arena was nested inside a PLA ring with infrared (IR, peak emission 850 nm) LEDs, which provided dark-field illumination of the animals without stimulating their photoreceptors. The ring also held a small thermometer (DS18B20, Maxim Integrated) positioned close to the arena and was placed on top a Peltier element with a thin layer of ASW underneath the agarose to improve heat conduction and image quality. Light stimulation was performed using LED illumination (green LED in NeoPixel LED array; emission 515–530 nm) and an IR filter (cut-of at 780 nm) positioned in front of the camera. Videos were recorded using an IR sensitive monochrome camera (DMK 33UP1300, The Imaging Source, Germany) and IC Capture software. An Arduino based circuit, interfacing with a GUI written in Python, provided stimuli and PID- temperature control.

### Recordings

**Figure 2:**
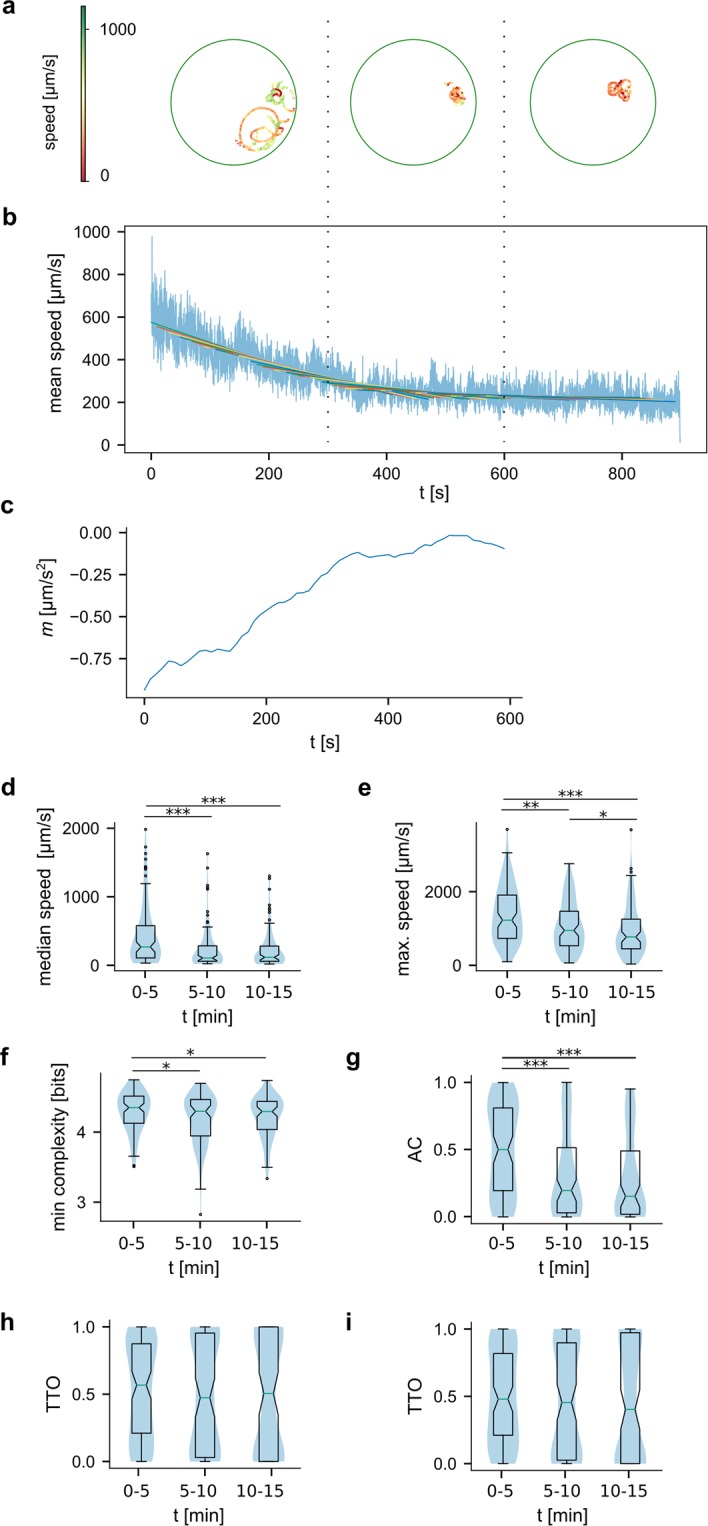
Acclimatization of animals to the arena. Analysis of acclimatization behaviour of larvae in the first 15 minutes after introduction to the arena. (**a**) An example trajectory of a path during the acclimatization period. The path is plotted in thirds, each corresponding to 5 minutes of the recording as illustrated by the dotted lines linking this and the next panel. (**b**) Average speed of 105 animals during the 15 minutes after introduction to the arena shows higher speeds immediately after the animals were exposed to the new environment. (**c**) Slope of linear regression fitted to the average speed (window 5 min, step size 10 s) shows the stabilisation of speed after ca 6 min. We compared (**d)** median speed, (**e**) maximum speed, (**f**) minimal reached path complexity, (**g**) activity coefficient, (**h**) TTO and (**i**) TDO between the first, second and last third of the 15 min acclimatization period (N_1_ = 105, N_2_ = 102, N_3_ = 92).

**Figure 3:**
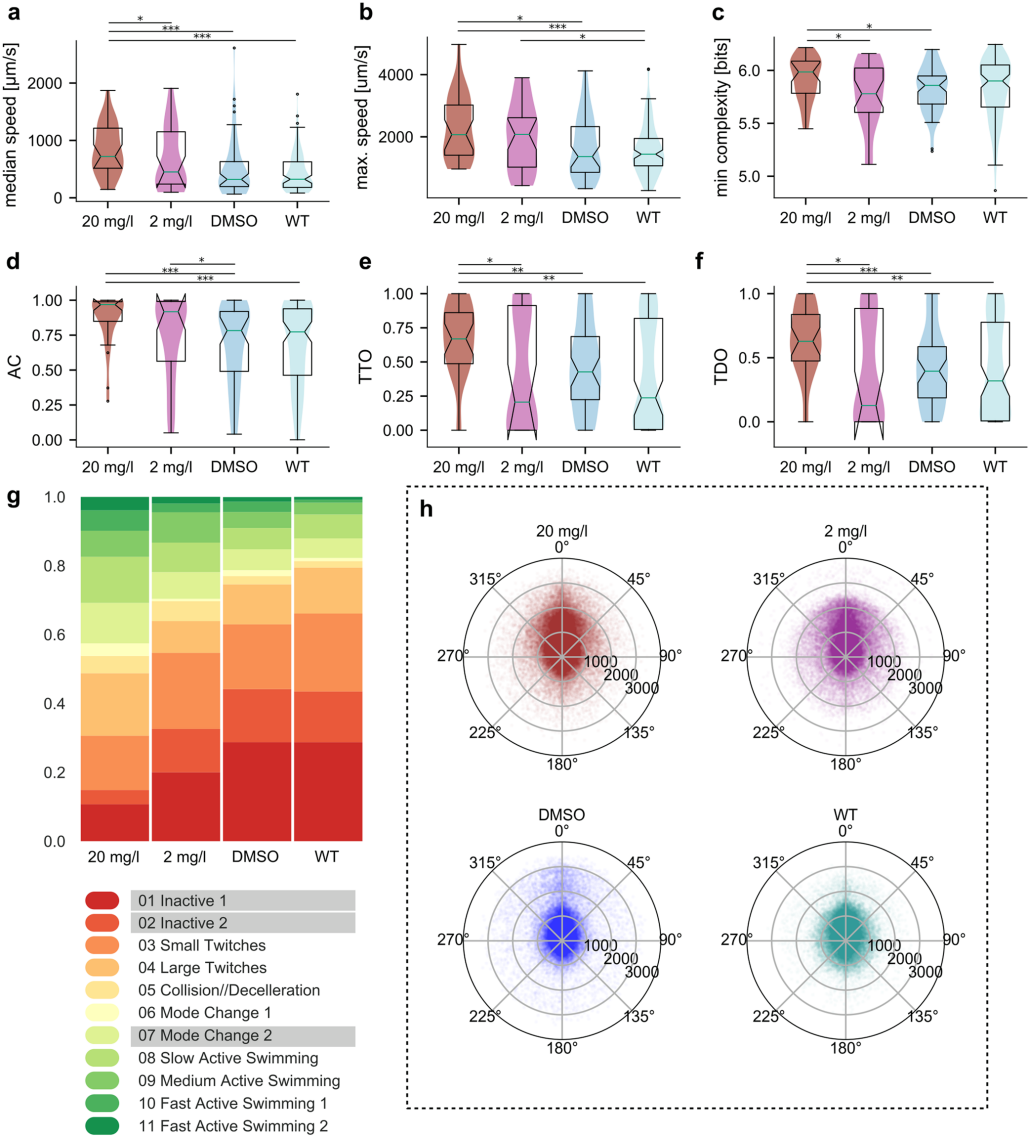
Thigmotaxis and the effects of modafinil on behaviour. Modafinil effects on (**a**) median speed, (**b**) maximum speed, (**c**) minimal path complexity, (**d**) activity coefficient and thigmotaxis measures (**e**) TTO and (**f**) TDO. The two groups affected by 20 mg/l and 2 mg/l modafinil are plotted in dark red and purple respectively, the control group in 0.1% DMSO in blue and wild type animals in light teal. (**g**) Distribution of behavioural modes for the groups. (**h**) Polar scatterplots of filtered speed values vs turn values the different groups. (N(20 mg/l) = 28, N(20 mg/l) = 27, N(DMSO) = 48, N(WT) = 101; number of points per polar plot is 100,000).

**Figure 4:**
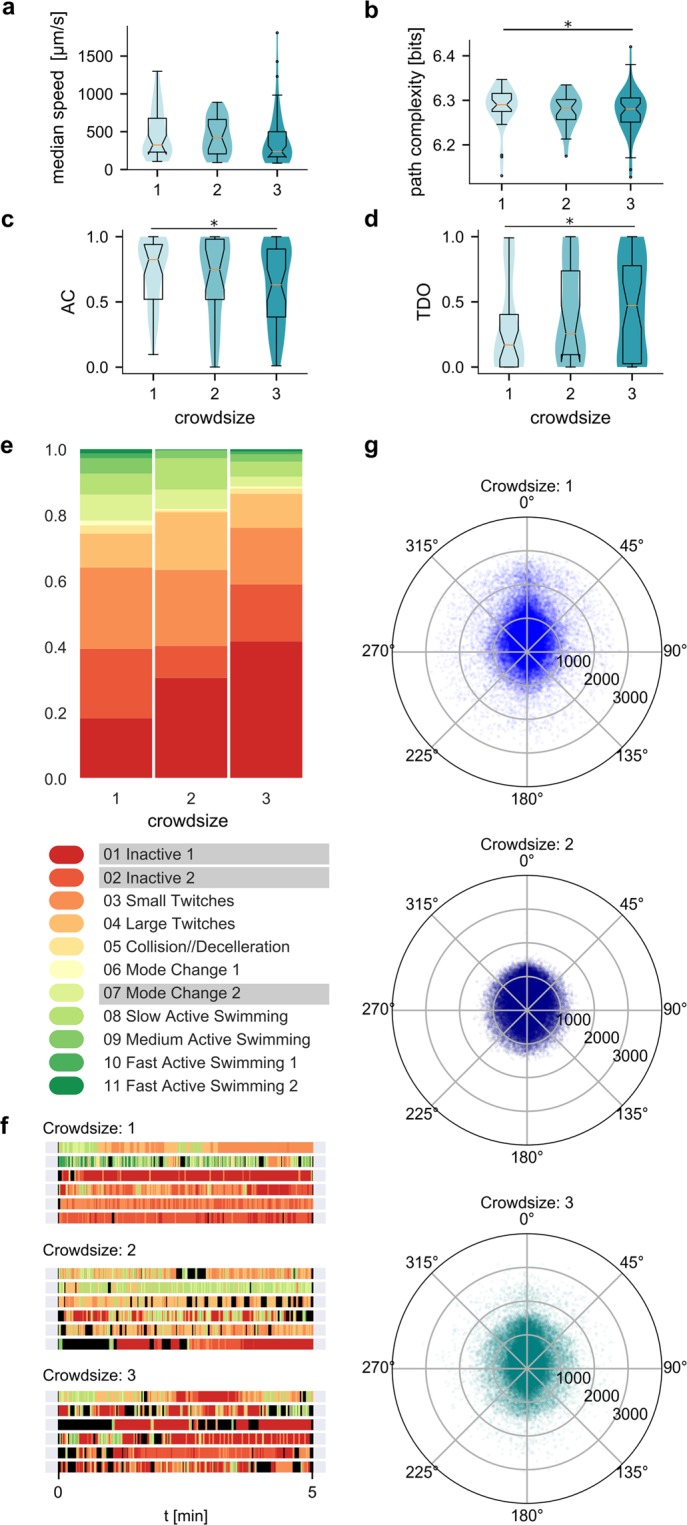
Crowdsize effects on behaviour. Crowdsize effects on (**a**) median speed, (**b**) path complexity, (**c**) activity coefficient and (**d**) TDO. (**e**) Distribution of behavioural modes in animals in experiments with different crowdsizes. The modes underlined in the legend are the biggest contributors to the Chi^2^ statistics (data in S1 Table). (**f**) Example ethograms of individual animals in crowdsize experiments. Each line represents a 5 min recording and is coloured based on the assigned behavioural mode, black colour represents missing frames where modes could not be assigned. (**g**) Polar scatterplots of filtered speed values vs turn values for different crowdsizes (N_1_ = 33, N_2_ = 22, N_3_ = 46; number of points per polar plot is 100,000).

**Figure 5:**
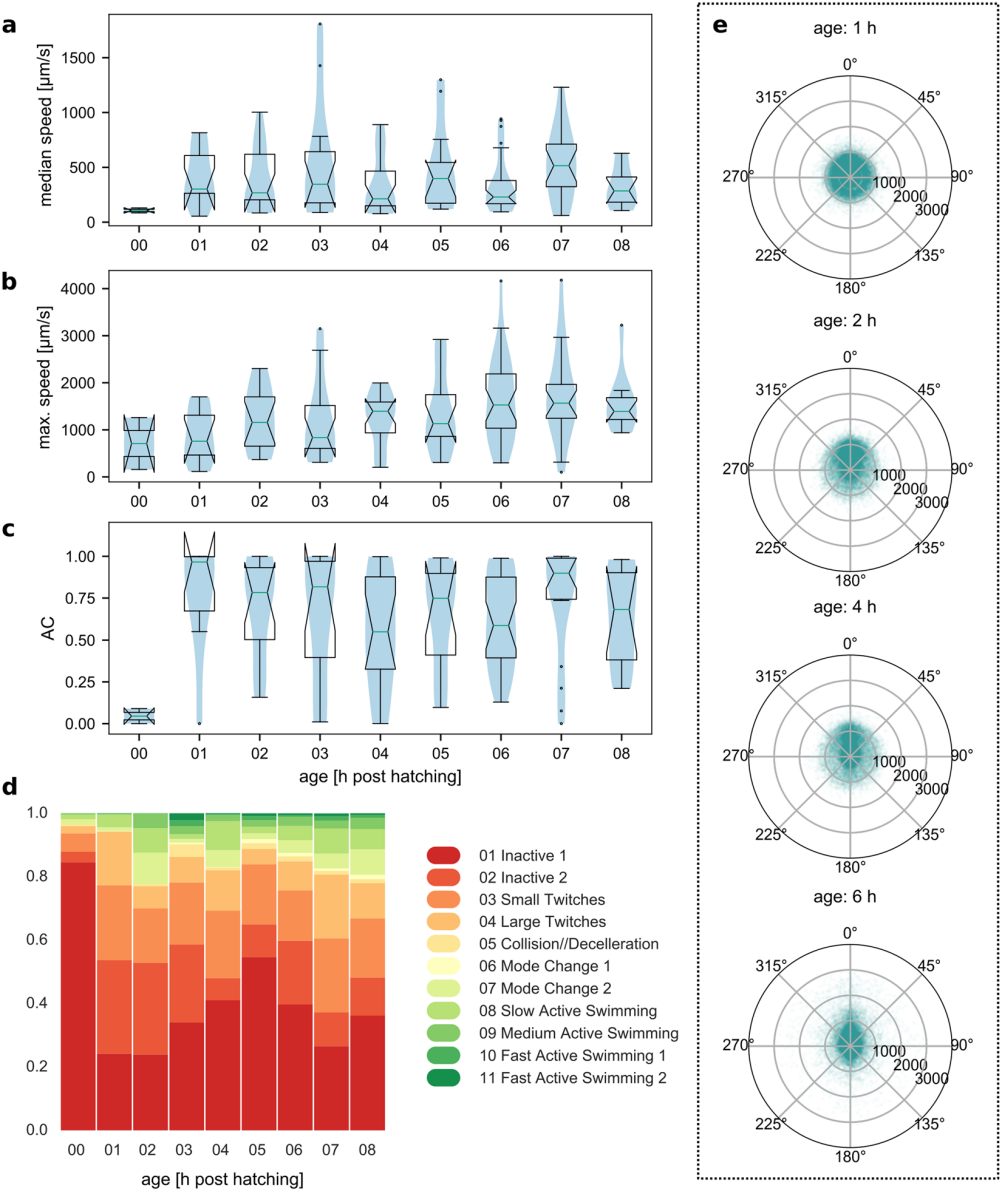
Changes in behavioural parameters due to post-hatching development. (**a**) Median speeds, (**b**) maximum speeds and (**c**) activity coefficient (AC) for WT animals reared at 14 °C at different age post hatching. (**d**) Distribution of behavioural modes in animals of different age. (**e**) Speed-turn plots for ages 1, 2, 4 and 6 h post hatching. (N(00) = 2, N(01) = 8, N(02) = 14, N(03) = 12, N(04) = 15, N(05) = 12, N(06) = 29,N(07) = 18, N(08) = 10, number of points per polar plot is 50,000).

**Figure 6:**
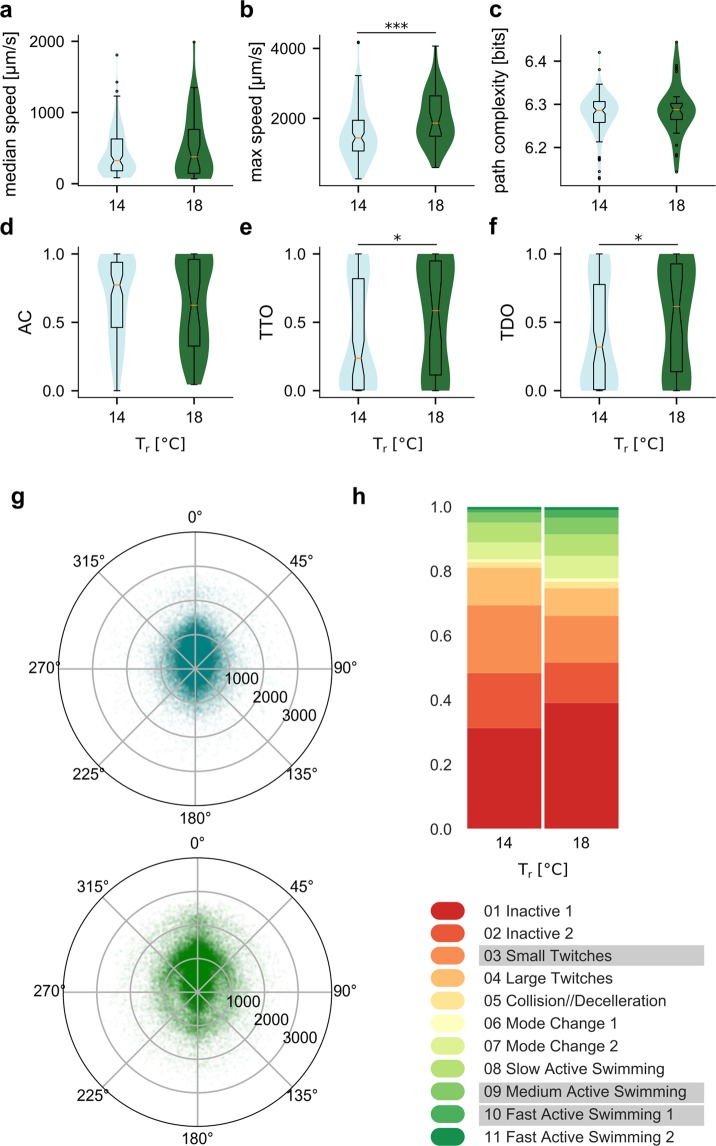
Effect of rearing temperature on behaviour. Rearing temperature effects on (**a**) median speed, (**b**) maximum speed, (**c**) path complexity, (**d**) activity coefficient and thigmotaxis measures (**e**) TTO and (**f**) TDO. (**g**) Polar scatterplots of filtered speed values vs turn values for animals reared and recorded at 14 °C (in teal) and at 18 °C (in green). (**h**) Distribution of behavioural modes for the two groups. The modes underlined in the legend are the biggest contributors to the Chi^2^ statistics (data in Supplemental Table 1) (N(14 °C) = 101, N(18 °C) = 36; number of points per polar plot is 100,000).

**Figure 7:**
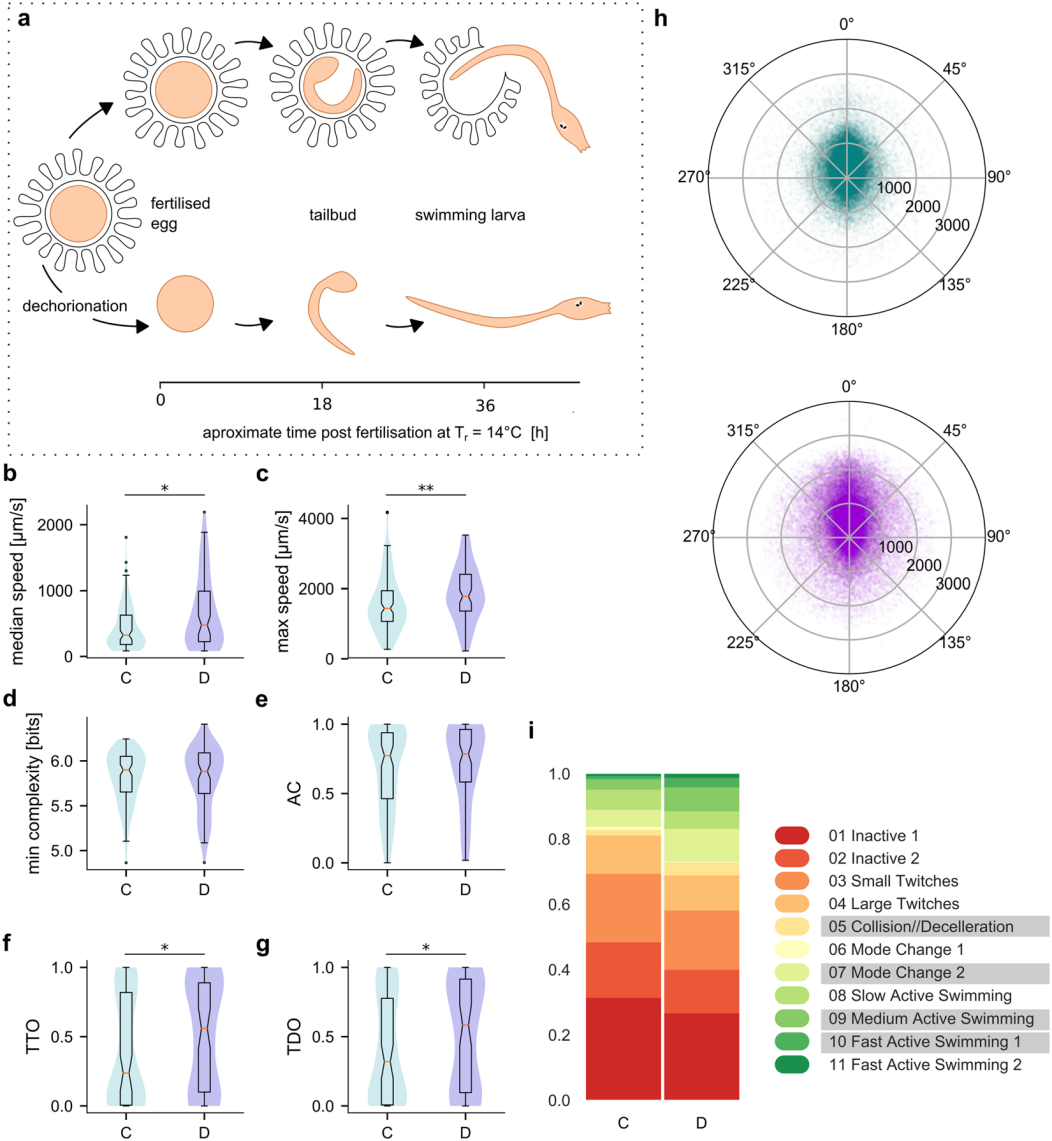
Effect of dechorionation on behaviour. (**a**) Schematic representation of the *C*. *intestinalis* embryo development inside the chorion (top) in untreated animals compared to dechorionation and subsequent development of a dechorionated embryo (bottom). We present dechorionation effects on (**b**) median speed, (**c**) maximum speed, (**d**) path complexity, (**e**) activity coefficient and thigmotaxis measures (**f**) TTO and (**g**) TDO. Chorionated animals are denoted as C, dechorionated as D. (**h**) Polar scatterplots of filtered speed values vs turn values for dechorionated (purple) animals compared to chorionated animals (teal). (**i**) Distribution of behavioural modes for the two groups. The modes underlined in the legend are the biggest contributors to the Chi^2^ statistics (data in Supplemental Table 1) (N(C) = 101, N(D) = 74; number of points per polar plot is 100,000).

We placed 1–3 animals in an agarose arena (10 mm in diameter and 3 mm high, approximate volume 236 mm^3^). Each animal in behavioural experiments was first filmed for a period of 15 minutes during acclimatization to the arena (at 10 frames/s). Subsequently we recorded 1 to 3 videos of 5 min duration at 30 frames/s to analyse either base line behaviour or the effects of light stimulation, rearing temperature or added drugs on behaviour. Therefore, we recorded each larva for a maximum of 30 minutes. The same larva was never used to record at different ages. For modafinil experiments, we first transferred the animals to a dish containing DMSO or modafinil and then immediately transferred them to the arena that also contained DMSO or modafinil (2 or 20 mg/l). We used data from animals with age range between 2 and 8 hours post hatching to obtain the plots presented in Figs 2–4 and 7. For Fig. 6, we used animals with age range between 2 and 8 hours post hatching for the 14 °C group while we used 0 to 3 hours post-hatching animals for the 18 °C group. For Fig. 5, we collected data from animals aged 0 to 8 hours post hatching. We list the n-values per age in Table S5. We would like to note that Figs 3, 6 and 7 share the same control data.

### Video conversion and analysis

Following acquisition the videos were analysed using the program ToxTrac^27^ which tracked the position of the animal as the centre of its detected shape. Prior to analysing, all frames for each video were enhanced with Contrast Limited Adaptive Histogram Equalization (CLAHE) with a clip limit of 1 and a tile grid size of 50 × 50 pixels. After histogram equalization, noise was reduced with a median blur with a tile grid size of 5 × 5 pixels. To input bright-background videos into the ToxTrac software, we inverted all frames by subtracting from a true white frame of equal size. Within the ToxTrac software, the ID algorithm used in our study was 2TCM sel. by Hist (MEE). We imaged approximately 710 animals and obtained around 1000 traces for further analysis and quantification.

### Behavioural parameters

Following ToxTrac analysis, all positional data was calibrated by taking into account the position and size of the recording arena and from the calibrated positions distances, speeds and subsequently all other parameters were derived.

Previous studies reported that *C*. *intestinalis* larvae modulate their locomotor activity levels through bursts of spontaneous activity^15,19,29^. We introduce a quantitative descriptor of locomotory activity, termed Activity coefficient (AC) and defined as the fraction of time an animal spent locomoting actively. We considered as active animals those exhibiting filtered speed values of 200 µm/s and above, which in practical terms includes all actively swimming animals as well as movement of the animal’s centre-point due to tail flicks and twitching (See Supplemental Fig. 1).

In our arena the assayed animals exhibited a large repertoire of trajectories while swimming in the arena that appeared qualitatively more or less “complex” to the observer (see Fig. 1e for example trajectories). We quantified the local path complexity using a method presented by Roberts *et al*.^30^. (See Supplemental text for detailed explanation of this method). As presented in Fig. 1e, the minimal complexity values calculated by this method correspond to the most predictable trajectory or, in other words, the most invariable movement in terms of represented speeds and directions in the time window.

We quantified thigmotactic behaviour by dividing the arena into two concentric zones with animals in the outer zone considered thigmotactic (Fig. 1d). With t = 1.45 mm the zones have equal surface area and the width of the thigmotaxis zone is above one animal body-length. The amount of thigmotaxis is quantified in two measures: “Total Time spent in Outer zone” (TTO) and “Total Distance travelled in Outer zone” (TDO), as utilised for zebrafish previously^31^. As an alternative measure for thigmotaxis we have used Median distance from the arena centre. Supplemental Fig. 9 displays median distance measurements for the data contained in Figs 2–4, 6 and 7.

All data analysis was performed with Python 3.5 using the numpy, pandas, scipy, scikit-learn and matplotlib libraries. We would like to note that for speeds under 500 µm/s, 180° points shown on the polar plots may be a result of a π/2 imprecision on the larval orientation. We excluded animals that were immobile and hence indistinguishable from dead from further analysis by excluding all traces where the maximal displacement from the starting position was less than one body-length (comprising approximately 10% of all examined traces). Similarly, traces where the animal was tracked for less than 2,000 frames were considered unrepresentative and excluded from further analysis. The tracked centre-point corresponded predominantly to the animal’s head. To filter out noise caused by the undulatory movement of the head during swimming the speed sequence values were filtered with a 1 Hz low-pass filter. All speed values presented are therefore filtered speeds and turn values refer to values calculated between coordinates 5 time-points apart for the same reason. (For detailed explanation on calibration and deriving of behavioural parameters, please see Supplemental text).

### Clustering and classifying of behavioural modes

Our final aim was to create a simple ontology of detectable behavioural modes in our data set that would serve as an additional tool in assessing the effects of different stimuli and genetic or pharmacological perturbations on the behavioural repertoire of the larvae. To this end, we performed unsupervised clustering of a minimal feature-set that describes the behaviour of the larvae. We created the feature set as follows:

For each used recording, the velocity vectors (ρ, θ) were calculated from coordinates 5 frames apart. Values ρ, Δρ and Δθ were used as measures for speed, acceleration and turns respectively. For each point the mean of a sliding window of [−25: +25] frames was used to include information of past and future movement. This resulted in a dataset of three features and ca. 1.8 million observations.

We next used an agglomerative clustering algorithm with ward-linkage to cluster the data set. While data distribution was not very discretised, we identified the point, at which further increasing the number of clusters would no longer reduce the total distance of all points to their respective cluster centre notably, which was at twelve clusters (Supplemental Fig. 7).

Using the clustered dataset, we trained a K-Nearest-Neighbours classifier and classified all collected traces. We found that apart from cluster 0 (accounting only for ca. 0.05% of the data) which turned out to result from instances where there were insufficient datapoints in the window for averaging, we could assign a relevant descriptor to each cluster according to its biological interpretation. We did this by inspecting the original videos and superimposing corresponding cluster data. When unable to resolve a biological difference between two clusters, we assigned them the same descriptive name. We list the behavioural modes in approximate order of speed of movement: 01 and 02 - Inactive 1 and 2, 03 - Small twitches, 04 - Large twitches, 05- Collision or Deceleration event, 06 and 07 - Mode change 1 and 2, 08 - Slow active swimming, 09 - Medium active swimming, 10 and 11 - Fast swimming 1 and 2.

(see Figure. 1g for some example traces of different modes, Supplementary Video 1–5, for an illustration of the speed and turn values present in the different clusters see data in Supplemental Fig. 2).

### Statistics

Whenever statistical significance was tested, we used nonparametric tests, since the datasets compared contained non-normally distributed data (See Supplemental Table 2 for results of Shapiro-Wilk normality tests), namely Levene’s test for equality of variances, Kruskal-Wallis analysis of variance and Mann–Whitney U test. All p values stated are using Mann–Whitney U if not otherwise stated. To compare the similarity of distributions in the scatterplots of speed vs turn values presented in this paper we compared the sorted Mahalanobis distances of these distributions (presented in Supplemental Fig. 3). Where relevant, we calculated the *χ*^2^ statistic for behavioural modes distributions and underlined the greatest contributor in the figure legend (see Supplemental Table 1 for *χ*^2^values). A detailed presentation of the n- values (animals, experiments and batches) is presented in the Supplemental Tables 3–5.

## Results

### Acclimatization to the arena

The introduction of an animal to a new environment, such as a tracking arena, is one of several potential triggers of generalised nervous system arousal^32–35^. Given that, arousal mechanisms are evolutionarily conserved^33,36–38^, we asked whether *C*. *intestinalis* larvae were subject to generalised nervous system arousal due to the transferring process to the tracking arena and the exposure to a new environment.

In order to answer this question, we decided that before recording the videos used for analysing baseline behaviour, we would record each animal for a 15 min period immediately after transferring the animals to the arena (Fig. 2). In the first minutes of the animal being exposed to the new environment its speed was generally higher (see Fig. 2a for example traces), which we quantified as the slope of linear regression over the average speed values of around 100 animals (Fig. 2b,c). From these results, we inferred that the animals adapted to the arena within approximately 6 minutes. We compared some basic behavioural parameters of individual animals between the first, second and last third of the 15 min acclimatization period (Fig. 2d–g). There are significant differences in both median speed (Fig. 2d, median at 267 µm/s in the initial 5 min vs. 101 µm/s in the second and 113 µm/s in the last 5 minutes; *p* (*1*. *vs*. *2*.) = 0.00009 and *p* (*0*.*1 vs*. *3*.) = 0,00004) and maximum speed of the animals (Fig. 2e, median at 1310 µm/s in the initial 5 min vs. 1003 µm/s in the second and 795 µm/s in the last 5 minutes*; p*(*1 vs*. *2*) = 0.0013, *p*(*1 vs*. *3*) = 0,000007, p(*2 vs*. *3*) = 0.0483). In addition, by plotting the average speed in the 5 minutes after the initial 15 minute acclimatization period we confirmed that average speed remained stable over longer periods of time (Supplemental Fig. 6). Interestingly, as a result of sensory arousal, *C*. *intestinalis* larvae exhibited higher minimum path complexity in the first 5 minutes after they are placed in the arena (Fig. 2f, 4.349 bit in the initial 5 min vs 4.297 and 4.293; *p*(*1*. *vs*. *2*.) = 0.0273, *p*(*0*.*1 vs*. *3*.) = 0.0038). This observation suggested that the animals followed a more unpredictable, or chaotic trajectory during the original acclimatization period. As they acclimatized, they followed trajectories exhibiting lower entropy and thus higher degree of predictability. In other organisms such as birds, the trajectory component showing higher entropy is associated with navigational uncertainty (66). Notably, the activity coefficient (Fig. 2g, median at 0.53 vs. 0.23 and 0.18; *p* (*1*. *vs*. *2*.) = 0.00011, *p* (*0*.*1 vs*. *3*.) = 0.000003) of the aroused animals is also significantly higher in the first 5 minutes, suggesting that bouts of inactivity are suppressed during this period.

### Thigmotaxis and effect of modafinil

During the acclimatization period to an open-field, mice perform a persistent exploration of their surroundings locomoting mainly along the walls of the arena. This behaviour is under the influence of the dopaminergic signalling and has been termed thigmotaxis^39^. We wondered whether *C*. *intestinalis* showed strong thigmotaxis during the initial acclimatization period to the arena. Contrary to the mouse findings, we did not observe any substantial difference in thigmotaxis between aroused larvae and animals that have acclimatized to the arena (Fig. 2h–i). Prompted by previous work in *C*. *intestinalis* that demonstrated the importance of dopamine for the modulation of larval photic responses^40^ we attempted to influence thigmotaxis in our animals by exposing them to an anxiotropic drug modafinil, which is a selective dopamine and norepinephrine transporter inhibitor^41,42^. In this set of experiments, we compared two groups of animals swimming in 20 mg/l and 2 mg/l solution of modafinil respectively to a control group in DMSO and to the untreated WT set (Fig. 3). We determined the dosage based on preliminary tests and previously published literature on modafinil effects on larval zebrafish^43^. The effect of 20 mg/l modafinil on thigmotaxis was very pronounced and statistically significant (Fig. 3e,f). While the median TTO value for the DMSO control was 0,427, it reached 0,669 in the 20 mg/l modafinil group signifying longer periods spent in the outer zone of the arena (*p* = 0.0015). Similarly, the TDO measure shows the modafinil-affected animals travelled much bigger proportions of their total distance in the outer zone of the arena (median TDO at 0.628 in the 20 mg/l modafinil group compared to the DMSO control at 0.395, *p* = 0.00099). For the 2 mg/l group the distribution for both thigmotactic measures was similar to the WT state and was not statistically different from either of the controls. It is important to note that DMSO alone seemed to affect the median distance from centre (Supplemental Fig. 9, DMSO vs none *p* = 0.113425) TTO (Fig. 3e DMSO vs WT untreated *p* = 0.133994) and TDO (Fig. 3f DMSO vs WT untreated *p* = 0.236986) relative to the untreated WT animals even though these effects were not statistically significant. Curiously, the two different concentrations of modafinil that we tested had opposite effects on the median distance from centre (Supplemental Fig. 9, 2 mg/l vs DMSO *p* = 0.170546, 2 mg/l vs WT untreated *p* = 0.113425) TTO and TDO (Fig. 3f, 2 mg/l vs DMSO *p* = 0.143451, 2 mg/l vs WT treated *p* = 0.257624). However, the observed effects between the lower concentration of modafinil (2 mg/l) and DMSO or WT untreated were not statistically significant.

The animals affected by modafinil also exhibited an overall more active set of behaviours with much higher representation of the active-swimming modes and less time spent inactively (Fig. 3g,h). This resulted in increased median and maximum speeds (Fig. 3a,b) and higher AC values (Fig. 3d) for animals in 20 mg/l modafinil. Path complexity (Fig. 3c) showed fewer differences with the only significant change being the higher minimal complexity values for 20 mg/l group (5.99 compared to 5.85 in DMSO, *p* = 0.0241; and 5.78 in 2 mg/l, *p* = 0.0352).

### Behavioural effects of larval crowd size

Across the animal kingdom, both direct and indirect interactions with conspecifics influence locomotion and response to environmental stimuli^44–46^. In the case of *Ciona* larvae there are good reasons to believe that the presence of conspecifics in the arena might change the individual behavioural metrics. Previous work has shown that large groups of larvae can form swarms upon mechanical or light stimulation^18,19^, that ascidian behaviours prior to settlement are largely influenced by conspecifics^24^, while the larvae exhibit a form gregariousness^47^.

In our study, we focused on the possibility of behavioural effects of small group interactions. To achieve this, we compared animals that were alone in the arena with animals recorded in pairs or groups of three. We found small differences in the basic behavioural parameters (Fig. 4). Crowd size did not affect median (Fig. 4a) or maximum speeds of movement substantially, but had a weak effect on the path complexity and activity coefficient (Fig. 4b,c). The average path complexity was lowest for animals in the crowd size 3 group (6.281 bits, compared to 6.290 bits for animals in crowd size 1 group, *p* = 0.05) and so was the median AC value (0.63 compared to 0.82 and 0.75 for animals in crowd size 1 and 2 groups respectively, *p*(*1*. *vs*. *3*.) = 0.045). We also found slightly more animals with higher thigmotaxis values, with the difference only significant for TDO (Fig. 4d) between crowd size 1 and 3 (median TDO is 0.17 for crowd size 1 vs. 0.47 for crowd size 3, *p* = 0.0227). Notably, crowd size showed no significant effects on the median distance from centre (Supplemental Fig. 2, 1vs2 *p* = 0.265304, 1vs3 *p* = 0.168713, 2vs3 *p* = 0.320831). The distribution of different behavioural modes shows higher representation of the less active modes corresponding to more time spent inactively in crowd size 3 animals (Fig. 4e, see Supplemental Table 1 for χ^2^ f values). In Fig. 4f, we present sample ethograms of 6 individual animals in different crowd size experiments. The smaller representation of higher speeds was also apparent when we plotted the distribution of speed values with the corresponding turn values on a polar scatterplot, presented for the different crowd sizes in Fig. 4g.

### Changes in behaviour due to post hatching development

Behavioural changes that result from aging have long been the focus of various senescence studies, but there are similarly important changes underlying the normal development of the animals’ behavioural repertoire through time^48–52^. *Ciona* larvae are subject to post-embryonic developmental changes and show behavioural modifications in their response to light and gravity^15,20^.

Here we examined the development of baseline behaviour in the early hours post-hatching. In animals reared and recorded at 14 °C, we observed that in the first hour after hatching they were mostly inactive, followed by a period of time when the larvae mostly twitched very actively and flicked their tails. Only at even later ages did we observe animals exhibiting longer bursts of active swimming behaviour. In Fig. 5, we show some of the basic behavioural descriptors compared between ages of WT animals reared at 14 °C. Both maximum and median speeds were lowest for animals immediately after hatching (Fig. 5a,b), accompanied by very low AC (Fig. 5c). While animals 1 h post fertilisation already achieved higher median speeds and generally had a very high AC (median at 0.992), their movement was less directional as can be seen by the distribution of turn values versus speed values (Fig. 5e) and the high representation of twitching modes in their behavioural repertoire (Fig. 5d). To minimise any potential skewing of the data because of changes due to development, we used animals of 2–8 hours post hatching age for all later comparisons, unless specified otherwise.

### Rearing temperature effects on behaviour and its ontogenetic development

Wild populations of *C*. *intestinalis* and *C*. *robusta* span a wide range of temperatures^53^. Our local animals develop best at lower rearing temperatures, possibly due to an adaptation to the cooler water temperatures in the Norwegian Fjords. We wondered whether rearing the animals at different temperatures would affect the robustness of their behavioural repertoire. To this end, we compared our wild type group reared and recorded at 14 °C to animals reared at 18 °C (a commonly used rearing temperature for *Ciona* in other labs). We describe the distinct difference in the speed of post hatching development at different rearing temperatures and the effects of temperature on behavioural repertoire (Fig. 6, Supplemental Figs 4, 5 and 10).

In our animals at 14 °C we can detect the first substantial, yet still weak, responses to the light-off signal at 6 h post hatching (Supplemental Figs 5 and 10), coinciding approximately with the period of higher locomotor activity in animals up to 8 h post hatching (Fig. 5). Animals at 14 °C continued to exhibit a light-off response with increased frequency and magnitude between 7 and 16 hours post hatching (Supplemental Fig. 10). For animals reared at 18 °C the light-off swimming response could be elicited between approximately 3.5 and 13 hours post hatching, at which time point when we stopped assaying animals (Supplemental Fig. 10). However, we note that *C*. *intestinalis* reared at 18 °C had a much narrower time window after hatching in which we could observe active swimming behaviour (in the absence of a sensory stimulus), with the majority of animals being highly inactive by age 4 h post hatching (Supplemental Fig. 4). We therefore only compared animals of age 0–3 h reared at 18 °C to the 14 °C reared animals of ages 2–8 h, since we assumed they correspond to the same post hatching development stages, with the animals reared at 14 °C exhibiting a slower post hatching development.

Animals that were reared as embryos and larvae at 18 °C and recorded at 18 °C still exhibited some differences compared to the ones at 14 °C (Fig. 6) assumed to be in the same developmental stage. Their traces were similar in median speed values (Fig. 6a) but reached significantly higher maximum speeds (Fig. 6b, median at 1864 µm/s for animals reared at 18 °C vs. 1440 µm/s for animals reared at 14 °C, *p* = 0.00084). The difference in AC (*p* = 0.3799) and path complexity (*p* = 0.4815) is not statistically significant (Fig. 6c,d), but there was a slight but significant effect on thigmotaxis (Fig. 6e,f). The median TTO for animals reared at 18 °C was 0.585 vs 0.237 at 14 °C (Fig. 6e, *p* = 0.035) and the median TDO at 18 °C was 0.616 vs 0.319 at 14 °C (Fig. 6f, *p* = 0.038). Our TDO and TTO measurements were not strongly corroborated by the median distance from the arena centre metric (Supplemental Fig. 9 *p* = 0.068229). At 18 °C we also observed a higher representation of medium-high speeds (around 1,000–1,500 µm/s) in combination with a wider range of turn values, while at lower speeds the variability of turns was smaller (Fig. 6g). This was matched with a lower representation of twitching modes and more occurrences of the modes representing swimming at medium speeds (Fig. 6h).

### Dechorionation effects

The eggs of *C*. *intestinalis* are nested in a chorion surrounded by follicle cells (Fig. 7a) and the normal development of left-right asymmetry in the embryo has been shown to be disrupted by the enzymatic removal of the chorion in a process termed dechorionation^54^. However, transient transgenesis of *C*. *intestinalis* via electroporation requires the dechorionation of the eggs. We therefore set out to test if dechorionation has specific effects on behaviour. Being aware of any potential effects of dechorionation will be vital for the future interpretation of behavioural phenotypes in transgenic animals. The dechorionated larvae achieved a higher median (Fig. 7b, 477 µm/s median vs 324 µm/s in chorionated animals, *p* = 0.0125) and maximum speeds (Fig. 7c, 1784 µm/s vs 1440 µm/s for chorionated animals, *p* = 0.0024). The differences in AC (*p* = 0.1997) and path complexities (*p* = 0.4477) were not statistically significant for our data (Fig. 7d,e). There was however a slight but significant effect on thigmotaxis (Fig. 7f,g), resulting in a higher median TTO (0.56 vs 0.23 for chorionated animals, *p* = 0.035), TDO (0.58 vs 0.32 for chorionated animals, *p* = 0.033) and median distance from the arena centre (Supplemental Fig. 9 *p* = 0.022593) values for dechorionated animals. The differences in distribution of turns and speeds were less apparent compared to the effect of temperature, but the increased representation of higher swimming speeds in dechorionated animals was evident in the polar scatterplots (Fig. 7h) as well as form the distribution of the behavioural modes (Fig. 7i). One could hypothesise that the observed differences in max. speed could reflect higher energy store levels in dechorionated animals, given that chorionated animals likely have to use up significant energy resources to release themselves from the chorion (which is absent in dechorionated animals). To test this hypothesis we performed Oil Red O stainings (Supplemental Fig. 8) to visualize neutral lipid contents of larvae and BODIPY 495/503 assays.

(Supplemental Fig. 8) in order to quantify and compare the neutral lipid contents of chorionated and dechorionated animals. We found that total volume of BODIPY 495/503 positive neutral lipid droplets was significantly higher (Supplemental Fig. 8 *p* = 0.00384, Box-Cox transformed, compared using an independent t-test) in dechorionated animals when compared to chorionated animals.

## Discussion

The potential of the *C*. *intestinalis* larva as an organism in which to perform neuroethological studies has been noticed for several decades. There have been a number of efforts to perform behavioural studies with increasing sophistication over the years^15–22,40^. In this study, we identified Toxtrac^27^ as a suitable open-sourced tracking software, we built customizable hardware and developed an automated behaviour analysis pipeline for *C*. *intestinalis* larvae.

By employing a clustering methodology, we classified the larval behavioural repertoire into 11 basic behavioural modes. These 11 modes provide an unbiased way to dissect the structure of behaviour and will allow us to perform a systematic classification of complex behavioural phenotypes that may result from pharmacological, genetic or optogenetic perturbations.

**Figure 8:**
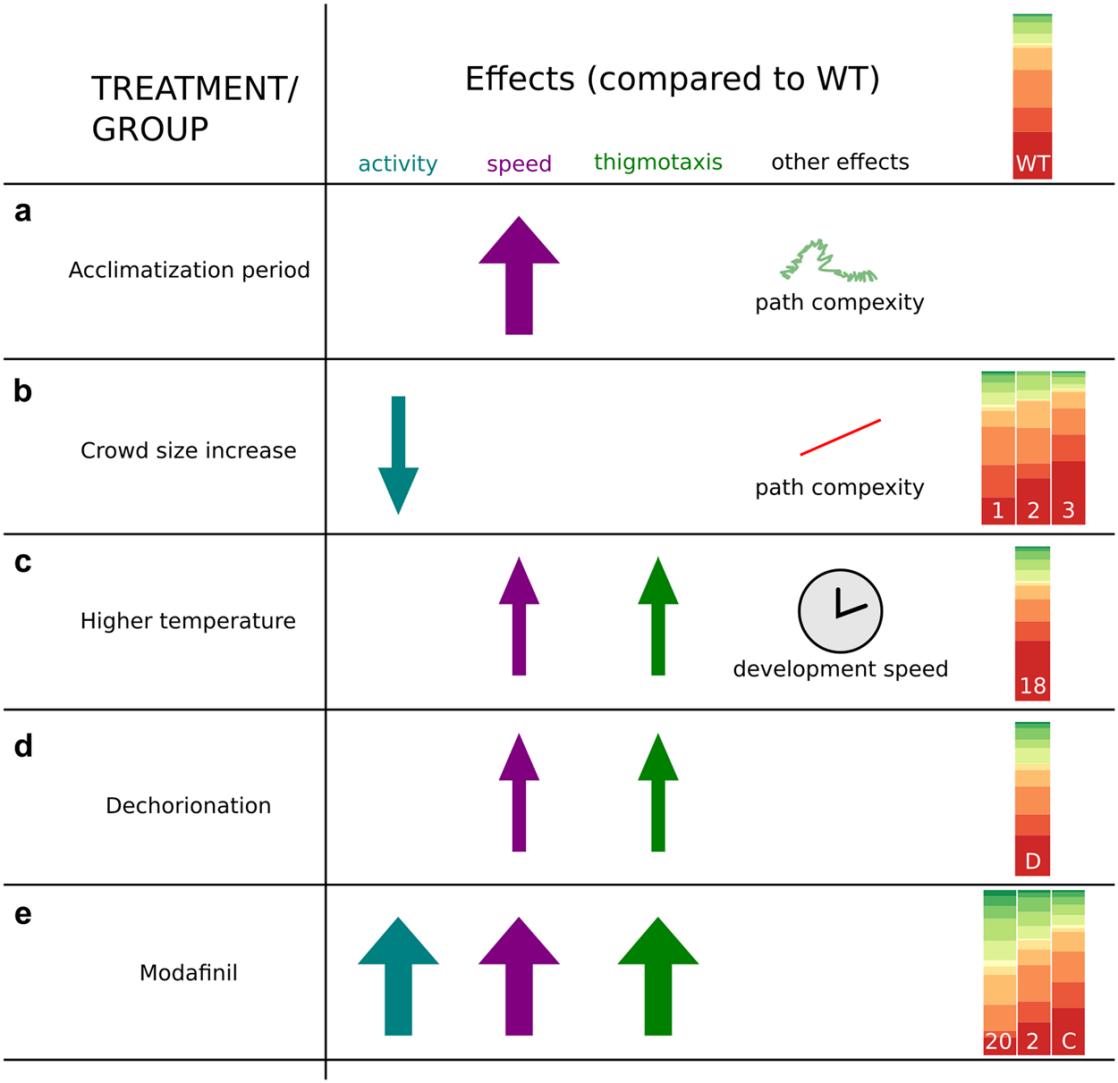
Summary. During the acclimatization period (**a**) *C*. *intestinalis* larvae exhibited sensory arousal which translated to higher speeds and increased path complexity. The presence of conspecifics in the arena (**b**) resulted in reduced locomotor activity, reduced path complexity and a change in the distribution of behavioural modes. We tested the robustness of behaviour in the context of rearing temperature (**c**) and dechorionation (**d**) treatments. Finally, the anxiotropic drug Modafinil (**e**) was able to modulate thigmotaxis, speed and the overall state of animal activity by changing the distribution of the behavioural modes.

The sensitivity of our measurements allowed us to quantify the activity levels of the animals in different experimental contexts. In accordance with our expectations, rearing conditions and external sensory cues seemed to influence the swimming strategy of the larvae. Interestingly, path complexity appeared to be modulated in opposite ways by sensory arousal and crowd size (Figs 2, 4 and 8). In agreement with previous reports, we found that larvae showed both bursts of activity and bouts in irregular intervals.

Our results indicate that *C*. *intestinalis* larvae enter a state of arousal during the first minutes after we place them in the arena (Figs 2 and 8). What might be the sensory circuits and molecular underpinnings of this phenomenon in C. intestinalis larvae? In the case of *C*. *elegans*, sensory circuits involved in sensing high threshold mechanical and noxious stimuli modulate arousal^34,55^. At the molecular level, work performed in *C*. *elegans* and zebrafish has highlighted the importance of neuropeptidergic and serotonergic signalling in modulating arousal^55–57^. We observed that the drug modafinil perturbs arousal in swimming *C*. *intestinalis* larvae. Given that modafinil acts as a selective dopamine^41^ and norepinephrine transporter inhibitor^42^, it is likely that monoamine signalling not only plays a role in modulating photic responses in ascidians^40^ but also modulates arousal.

Using an open-arena to monitor our animals, we noticed that a fraction of larvae exhibited strong thigmotactic behaviour. This appears to be an adaptive behaviour that is evolutionarily conserved, where the circular wall of the arena allows the animals to exhibit a defensive response (i.e. to hide from potential predators) and facilitates their orientation in space^58^. The ecological usefulness of the thigmotactic behaviour exhibited by *C*. *intestinalis* larvae is unclear. However, it has been hypothesised that thigmotaxis, amongst other behaviours, may be involved in the selection of habitats for larval settlement^24^. Moreover, we found that modafinil increased thigmotaxis levels in *C*. *intestinalis* larvae (Figs 3 and 8). This is interesting in light of the fact that modafinil can reduce thigmotaxis levels in zebrafish^43^. The strong effect that the anxiotropic drug modafinil has on *C*. *intestinalis* larval thigmotaxis is evidence that a common mechanism might mediate thigmotaxis across taxa^43,59^. Future work should explore the molecular and cellular underpinnings of thigmotactic behaviour in *C*. *intestinalis* larvae and aim to understand the ecological context in which it may be used.

In this study, we quantified how progression through larval life changes the behaviour of the animals, and found age dependent differences in the distribution of behavioural modes (Figs 5 and 8). We would like to note two confounding parameters for our conclusions. Firstly, that when the numbers of animals assayed is relatively small then the individual heterogeneity in behaviour may have a strong influence on our overall conclusions. A second confounding parameter is that a very large fraction of previous studies used *C*. *robusta* or C. savignyi larvae and thus direct comparisons between our experiments and those reported elsewhere might be hard to make. For example, developmental timing differences and/or species specific variations in nervous system structure and function may affect how or when two different species perform hallmark behaviours as discussed recently by Salas *et al*.^22^. Nonetheless, our data supports previous findings showing that light dependent behaviours change during the larval life^15,19,20^. What are the cellular and molecular mechanisms that bring about these changes? One possibility is that some neurons of the larval system fully differentiate and connect to the nervous system only after hatching. Indeed, there has been evidence for post embryonic terminal differentiation of dopaminergic cells in *C*. *intestinalis* larvae^60^. The authors of this study postulated that dopamine might modulate the neural circuits involved in the age-dependent changes in swimming behaviour of the larva. A large number of animals exhibit behavioural changes linked to post-embryonic development of their nervous system. For example, in *Xenopus laevis*, locomotor activity patterns are modified as the animals transition from sessile hatchlings to free-swimming larvae^51^, through changes in the cell properties of neurons^50^ and a nitrogen oxide signalling mechanism^49^. Other examples of age dependent behavioural changes include motor behaviours in zebrafish^48^ and larval Angelfish^52^. We believe that the use of a fast-growing larval animal with associated changes in body proportions and shape provides an excellent opportunity to understand how alterations in biometrics and sensory capacity may relate to simultaneous changes in locomotory behaviour.

In the present study we performed a series of experiments aimed at testing the robustness of the larval behavioural repertoire in different contexts including rearing conditions, age and crowd size.

Our data show that when we perturb rearing conditions such as cultivation temperature and removal of the chorion the behavioural metrics that we used in our analysis showed limited but substantial effects in larval behaviour. In particular, we found that main effect of a higher rearing temperature was an increase in the post-hatching development of the larva. This is something that is reasonable given that temperature is a known modulator of key physiological processes^61,62^ and behaviour in numerous organisms^63–65^. However, we note the higher representation of medium-high speeds in combination with a wider range of turn values (Figs 6 and 8) observed in animals reared at 18 °C. When compared to previous studies performed at 18 °C, such as the elegant study by Nakagawa *et al*.^17^ we find that our maximal speed values are similar but our median speeds are lower. These differences may be due to the fact that we quantify median speed values over 5 minutes, which are lower due to long periods of inactivity, compared to quantifications of average speed values over 15 seconds of swimming^17^. Furthermore, in light of the recent clarification of the relationships within the *Ciona* genus^66,67^ it is likely that some of the of observed differences may have to do with the possibility that our study is using a different species (*C*. *intestinalis*) compared to previous studies which may have in fact used *C*. *robusta*.

Our comparison of chorionated versus dechorionated animals revealed differences in speed and thigmotaxis. A notable result that we were surprised to find is that dechorionated animals showed higher speeds compared to chorionated animals (Figs 7 and 8). This was surprising because dechorionation perturbs the establishment of ascidian brain asymmetry^68^ and therefore we might have expected that dechorionated larvae might be worse swimmers. There are a number of possible interpretations for this observation. One possibility is that the tunic is partially formed as a result of the dechorionation^69^ and this may modify sensory inputs to the nervous system thus locking the animal in a sensitized “high activity” state. Another possible explanation is that, according to previous work, the larval tunic forms “blades” which likely play a role in the hydrodynamics of the swimming larva. The dechorionated animals might somehow have obtained a hydrodynamically more favourable configuration under our dechorionation conditions. Finally, in light of the neutral lipid analysis, it is very likely that the speed differences may be, at least in part, due to higher energy stores present in dechorionated animals.

When animals must locomote in crowded conditions, it is advantageous to be able to sense other conspecifics as well as their environment. This may result in avoiding collisions and possibly in coordinated behaviour. Our data demonstrates that the presence of two or three animals in the arena can alter an individual’s behavioural metrics, including an enhancement of thigmotaxis in the presence of multiple individuals. In light of previous work performed in *C*. *intestinalis*, it will be interesting to determine if and how *C*. *intestinalis* larvae achieve coordinated movement, especially in the presence of sensory stimuli. The combination of our hardware with the ToxTrac software^27^, which allows for solving occlusions events when tracking multiple animals^70^, is a suitable platform for future experiments.

Our current automated image based tracking approach is relying on marking each animal with a centroid rather than segmenting out the entire shape of the animal in order to generate an outline or a skeleton. We therefore are lacking postural information that would enrich our dataset significantly. This presents an important next step towards obtaining a complete ethogram of *C*. *intestinalis* larval behaviour. Furthermore, we are testing our animals in an open field arena that is suitable for recording a relatively small number of animals, possibly in a setting that is relatively distant to the natural ecological niche of the larvae. Numerous experimenters who are trying to obtain high quality tracking data in a controlled environment face this problem^3^. We envision that in the future the use of larger arenas and the ability to deliver multiple sensory stimuli, reaping the benefits of the open architecture of the behavioural setup, will allow us to study other ecologically relevant behaviours such as settlement behaviour and metamorphosis more closely. At the same time given the large numbers of Ciona larvae available at any one time, the cheap and expandable nature of the hardware in combination with the open source software we envision that performing inexpensive medium and high-throughput pharmacological screens using behavioural phenotyping is now well within reach.

## Supplementary information

Supplementary Information

S1 Video Behavioural mode 1

S2 Video Behavioural modes 2 and 3

S3 Video Behavioural modes 3, 4 and 8

S4 Video Behavioural modes 1, 3, 4, 7, 8 and 9

S5 Video Behavioural modes 5, 7, 9, 10 and 11

## Data Availability

We have deposited the designs for the behavioural setup components that were 3D printed here: https://github.com/ChatzigeorgiouGroup/Rudolf-Dondorp-2018/tree/master/3d%20Cad%20files. The dataset used for this study is available here: 10.5281/zenodo.1298978. The analysis code is located in our GitHub repository: https://github.com/ChatzigeorgiouGroup/Rudolf-Dondorp-2018/tree/master/Code.
