# Supplementary material for: Automated behavioural analysis reveals the basic behavioural repertoire of the urochordate Ciona intestinalis
Source: Sci Rep. 2019 Feb 20;9:2416. doi: 10.1038/s41598-019-38791-5 (PMC6382837; doi:10.1038/s41598-019-38791-5)
Supplement: Supplementary file 1 — Supplementary Information [file 41598_2019_38791_MOESM1_ESM.pdf]

Supplementary Information

"Automated behavioural analysis reveals the basic behavioural repertoire of the urochordate *Ciona intestinalis*"

Jerneja Rudolf[1]#, Daniel Dondorp[1]#, Louise Canon[1,2]§, Sonia Tieo[1,3]§, Marios Chatzigeorgiou[1]*

# These authors contributed equally to this work. § These authors contributed equally to this work.

[1] Sars International Centre for Marine Molecular Biology, University of Bergen, Thormøhlensgate 55, 5006 Bergen, Norway. [2]École Nationale Supérieure de Chimie de Montpellier, 240 Avenue du Professeur Emile Jeanbrau, 34090 Montpellier, France, [3] University Paris Diderot-Paris7, 5 rue Thomas Mann, 75013 Paris, France

* Marios.Chatzigeorgiou@uib.no

Supplemental information on used methods

For every analysed video the position of the centre of the arena is determined with a Hough Circle Transform algorithm in OpenCV in Python. For every trace the [x,y]-positions are corrected so that [0,0] was at the centre of the arena. All positions were then multiplied by the factor of 11.56µm/pixel for the setup the recording originates from. From these positions distances, speeds and subsequently all other parameters are derived. We excluded animals that were completely immobile and hence indistinguishable from dead from further analysis by excluding all traces where the maximal displacement from the starting position was less than one body-length (comprising approximately 10% of all examined traces). Similarly, traces where the animal was tracked for less than 2,000 frames were considered unrepresentative and excluded from further analysis.

Activity coefficient (AC) is defined as the fraction of time an animal spent locomoting actively. Filtered speed values of 200 µm/s and above were considered as active. In Supplemental Figure 1 we present the distribution of speed values in our wild-type animals. Speeds higher than the node in the distribution at ca. 200 µm/s in practical terms represent active swimming as well as movement of the animals' centre-point due to tail flicks and twitching, but exclude moments of complete immobility or very slow drifting due to inertia.

Local path complexity was calculated using a method presented by Roberts et al [30]. In summary, this method uses embedding matrices for positions in a specific time window, over which the local path complexity is calculated in bits of entropy. At each time point we consider a matrix of (x,y) positions over 30 frames and by subtracting the mean values for x and y we are focusing on the variation of position around the mean in the selected time window. We perform singular value decomposition of this embedded matrix and calculate entropy of the normalised eigenvalues. Minimal complexity values calculated by this method correspond to the most invariable or 'predictable' trajectory with a straight-line trajectory resulting in the lowest value and paths with variation in speed and direction of movement resulting in high values. It is noteworthy that this measure is independent of the absolute values for mean position, speed and orientation in the time window, so that local path complexity represents the trace's variability at a given period regardless of the mean speed of movement.

BODIPY 493/503 staining of neutral lipids

Newly hatched chorionated or dechorionated animals were fixed in 4%PFA for one hour at room temperature. Then animals were washed in 1x PBS three times for 15 minutes each time. Animals were permeabilized with Proteinase K (4µg/ml for 30 minutes at 37°C. Proteinase K activity was stopped with Glycine (2mg/ml) for 5 minutes. The animals were washed three times in 1x PBS. Each wash lasted 5 minutes. We postfixed with 4%PFA for 1 hour at room temperature. Subsequently animals were incubated in BODIPY 493/503 (Thermo Fisher D3922) at a concentration of 1mg/ml dissolved in DMSO. The incubation lasted for two hours. Animals were then washed in 1x PBS three times for 15 minutes each time. A final 1 hour long incubation in 1x PBS with 1/10,000 DAPI was performed to stain cell nuclei. The PBS/DAPI solution was replaced with a DABCO/Glycerol mix and animals were mounted on slides for confocal imaging.

BODIPY Image analysis

Confocal stacks (40x) of dechorionated and wild type animals treated with BODIPY 493/503 were obtained on a Leica SP5 confocal microscope using equal settings for all acquisitions. Using FIJI/ImageJ scripts all acquired stacks were analyzed by splitting out the channel containing the BODIPY signal and calculating statistics on the volumes found by the built-in “3D objects counter” (parameters: min.=10, max.=1000. threshold.=125, exclude_objects_on_edges). This data was subsequently analyzed and visualized using python (numpy, pandas, matplotlib and scipy.stats). For each measured animal the total volume of all present signal was calculated. Data was transformed using a Box-Cox transformation, tested for normality with a Shapiro-Wilk test and compared using an independent t-test.

Oil Red O staining

Stock solution of Oil Red O (Sigma O0625) was prepared as follows : We added 0.5g of Oil Red O to 100ml of isopropanol and shaked for several hours. To generate a working solution, we 40ml of stock solution and diluted this to 60% using H_2O, shaked for >1hr and filtered using a o,22µm filter. Chorionated and dechorionated larvae were harvested and fixed in 4%PFA for 1 hour at room temperature. Then they were washed 3 times in 1xPBS (15 minutes each wash). The animals were then placed in 60% isopropanol. After one hour the isopropanol was replaced with Oil Red O and animals were shaken for 24 hours. The animals were then washed 3 times in 1x PBS/0,01% Triton over a 2 hours period, prior to be mounted on a slide for microscopic analysis.

Supplemental results and statistical data

S1 Table: Table of all relevant χ^2 values for the distributions of behavioural modes in different experiments.

Modes	Modafinil groups (AD Fig 3)	Crowdsize groups (AD Fig 4)	18°C vs 14°C reared animals (AD Fig 6)	Dechorionated vs.WT (AD Fig 7)
01 Inactive 1	23714	12247	1893	429
02 Inactive 2	14669	4996	3062	369
03 Small Twitches	4336	2389	8497	2548
04 Large Twitches	7052	4394	1556	1595
05 Collision//Deceleration	11882	2436	513	4641
06 Mode Change 1	8994	3238	374	2341
07 Mode Change 2	8612	6543	3121	6894
08 Slow Active Swimming	9766	4155	268	1065
09 Medium Active Swimming	12230	3712	5345	8327
10 Fast Active Swimming 1	18216	1526	6884	6641
11 Fast Active Swimming 2	9531	1392	445	718
Summed χ^2 statistic	129003	47029	31959	35569

	Shapiro-Wilk test p values					
datasets	median speed	max. speed	path complexity	AC	TTO	TDO
Aclimatization datasets (Fig 2)						
1st 5 min	0.00000	0.00042	0.00222	0.00000	0.00000	0.00000
2nd 5 min	0.00000	0.00086	0.00002	0.00000	0.00000	0.00000
3rd 5 min	0.00000	0.00000	0.00038	0.00000	0.00000	0.00000
WT dataset (Fig 3, 6, 7)	0.00000	0.00028	0.00006	0.00000	0.00000	0.00000
Modafinil datasets (Fig 3)						
20 mg/l modafinil	0.32251	0.07103	0.03483	0.00000	0.02120	0.09242
2mg/l modafinil	0.00237	0.11647	0.01489	0.00011	0.00003	0.00004
DMSO	0.00000	0.01036	0.12353	0.00012	0.02320	0.00863
Crowdsize datasets (Fig 4)						
Crowdsize 1	0.00176	0.03720	0.00009	0.00085	0.00001	0.00001
Crowdsize 2	0.55994	0.00043	0.33552	0.00333	0.00160	0.00332
Crowdsize 3	0.00000	0.27685	0.00883	0.00182	0.00005	0.00016
18C dataset (Fig 6)	0.00019	0.39978	0.04430	0.00085	0.00010	0.00025
Dechorionated dataset (Fig 7)	0.00000	0.09165	0.00001	0.00000	0.00000	0.00000

S2 Table: Shapiro-Wilk p-values for all datasets

Low p (<0.05) values indicate that the dataset is not sampled from a normal distribution, justifying the use of non-parametric statistical test in testing significance of differences between datasets.

Since in all comparisons there is at least one non-normally distributed dataset we used the non-parametric Mann-Whitney U test throughout, except for the BODIPY experiments where a t-test was used.

S3 Table: Collective information for n-values (animals, experiments, batches)

experimetal conditions	N(animals)	N(experiments)	N(batches)
Adaptation	105	51	35
WT at 14°C	101	69	18
Dechorionated	74	53	10
WT at 18°C	36	35	10
Modafinil 2mg/l	27	24	2
Modafinil 20mg/l	20	16	3
DMSO	37	30	4

S4 Table: Crowdsize experiments n-values (animals, experiments, batches)

crowd size for WT at 14°C	N(animals)	N(experiments)	N(batches)
1	33	29	7
2	22	16	7
3	46	27	10

S5 Table: Hours post hatching at 14°C n-values (animals, experiments, batches)

age[h p.h.] in WT at 14°C	N(animals)	N(experiments)	N(batches)
0	2	2	1
1	8	7	2
2	14	10	4
3	12	7	4
4	15	8	5
5	12	10	8
6	29	23	10
7	18	12	4
8	10	8	4

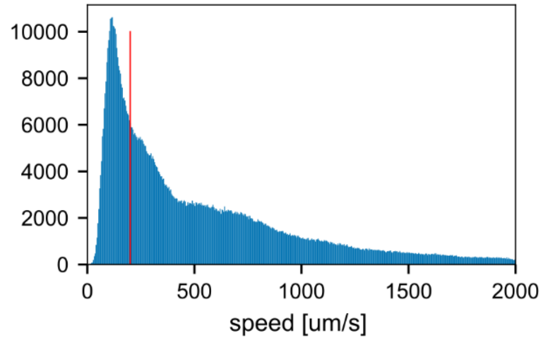

Supplemental Figure 1. Speed distribution for wild-type animal.
Histogram of filtered speed values for all 149 animals at 14°C and not influenced by drugs, dechorionation or light stimulation. Speed values are binned into 1,000 bins and only plotted up to 2,000 µm/s to allow better insight into the distribution at lower values. The red line indicates our chosen cut-off at 200 µm/s used for calculating the activity coefficient (AC).

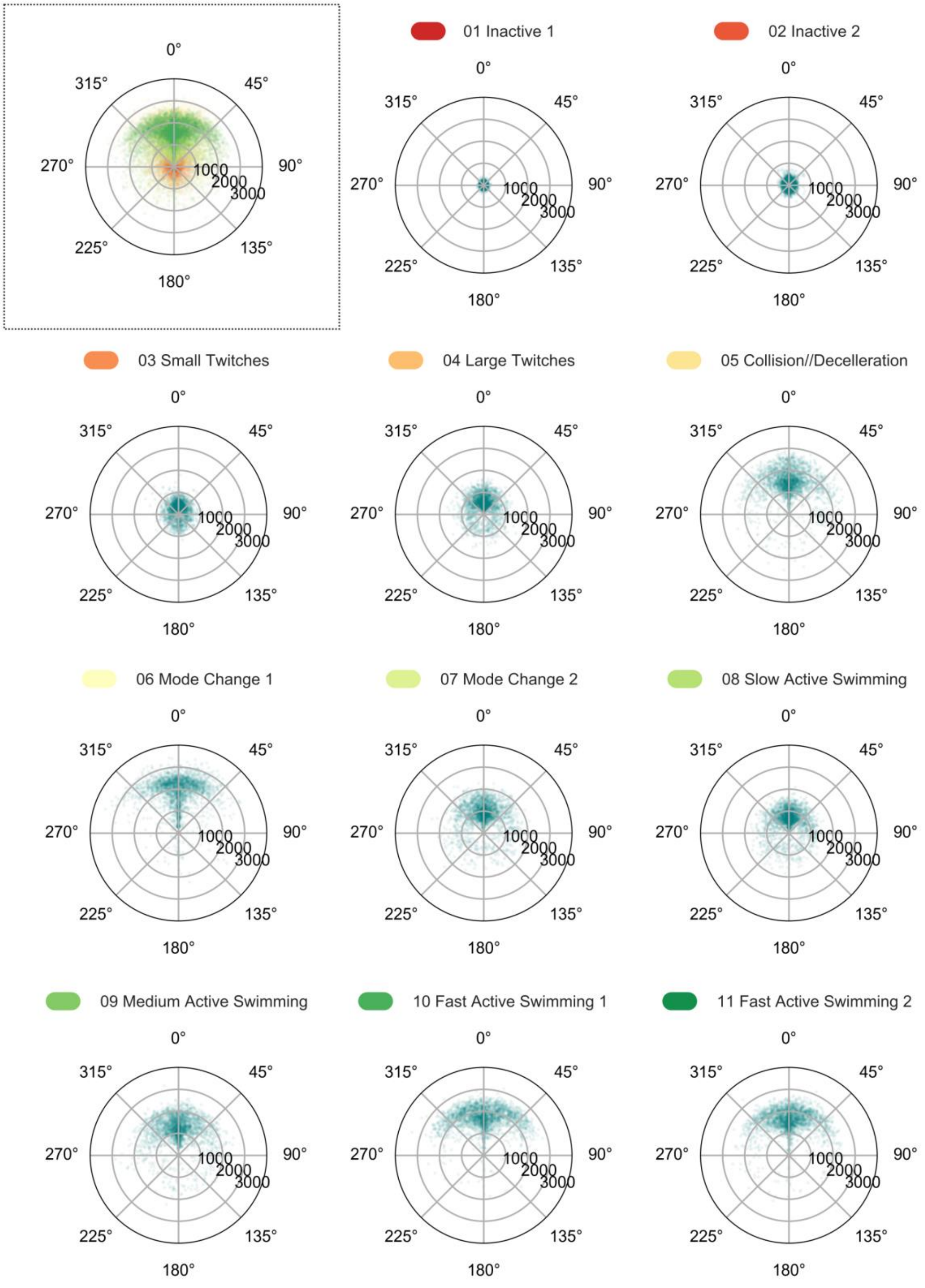

Supplemental Figure 2. Representation of turn and speed values in different behavioural modes
Speed and turn values at time points classified into different behavioural modes as categorised in the methods (2,600 randomly-sampled points per plot). Inside the dotted line, all the polar point-clouds are superimposed and plotted in the respective colours used to represent the modes throughout the papers figures.

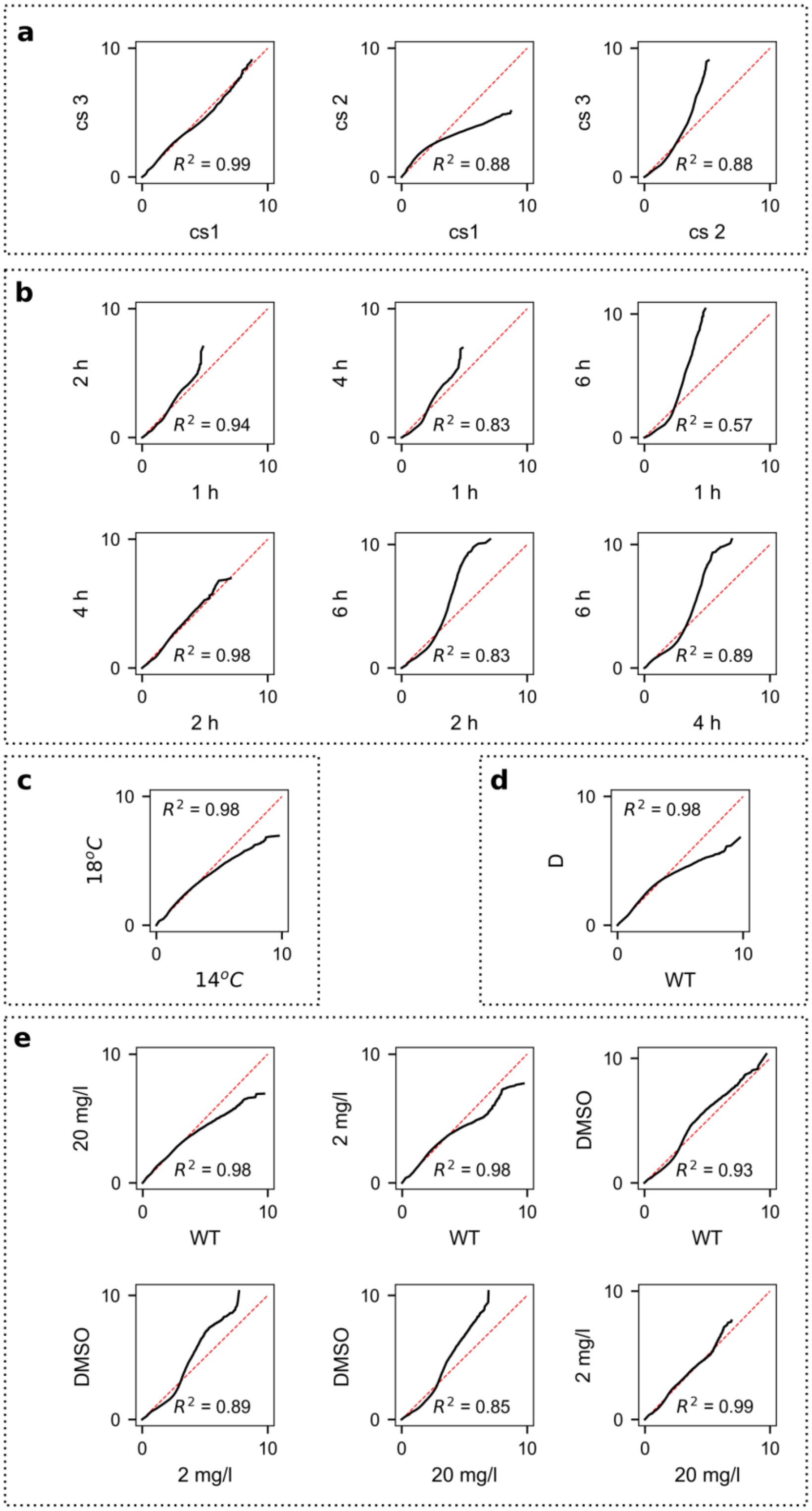

Supplemental Figure 3. Mahalonobis distance statistics

Mahalonobis distances and R^2 values for the speed-turn polar plots in the following order: **(a)** corresponding to Fig. 4g, **(b)** corresponding to Fig. 5e, **(c)** corresponding to Fig. 6g, **(d)** corresponding to Fig 7h, **(e)** corresponding to Fig. 3h.

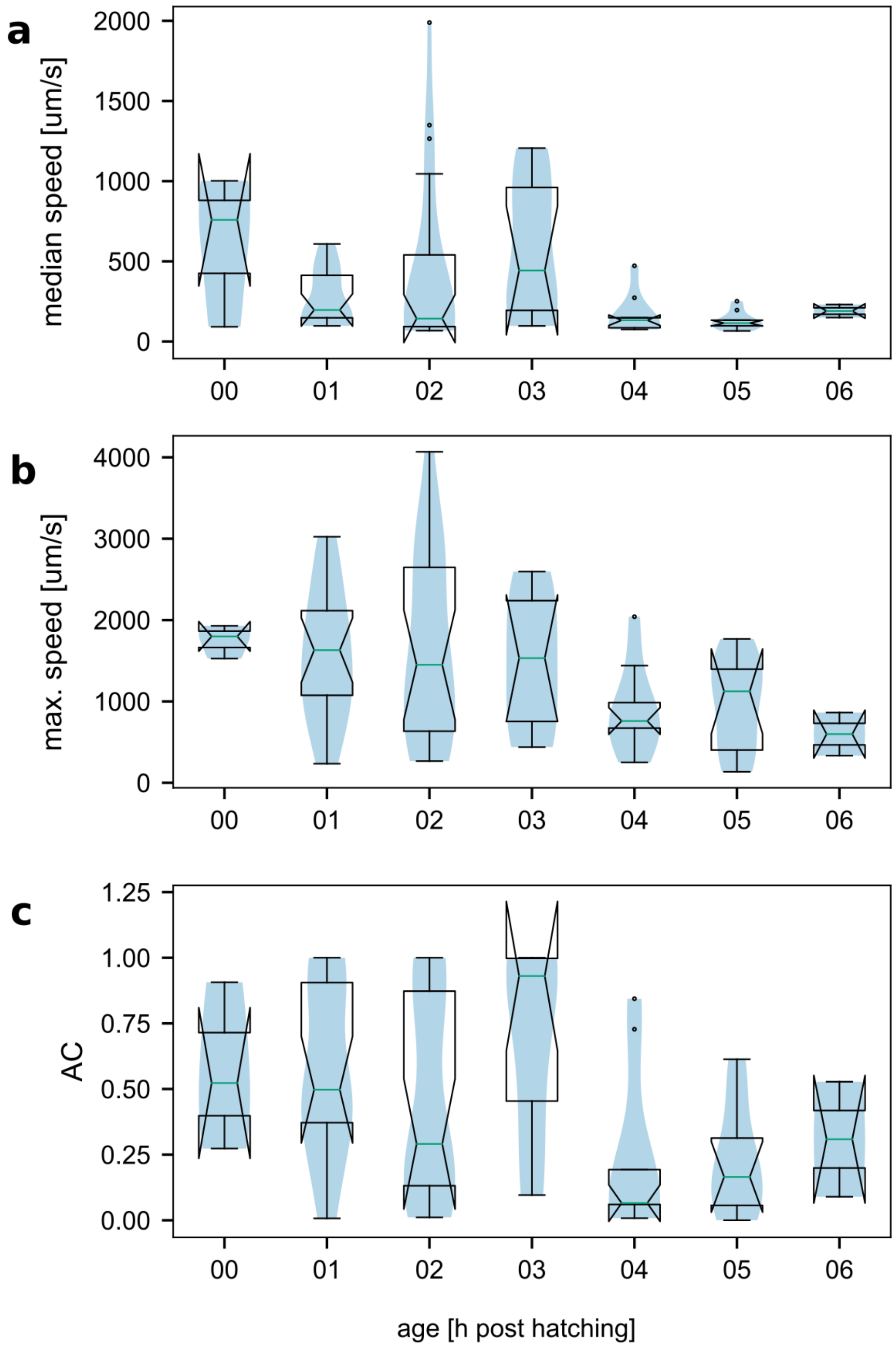

Supplemental Figure 4. Age dependent changes in animals reared at 18°C

(a) Median speeds, **(b)** maximum speeds and **(c)** activity coefficient (AC) for WT animals reared at 18°C at different age post hatching (N(00)=3, N(01)=17, N(02)=22, N(03)=9, N(04)=9, N(05)=9, N(06)=2).

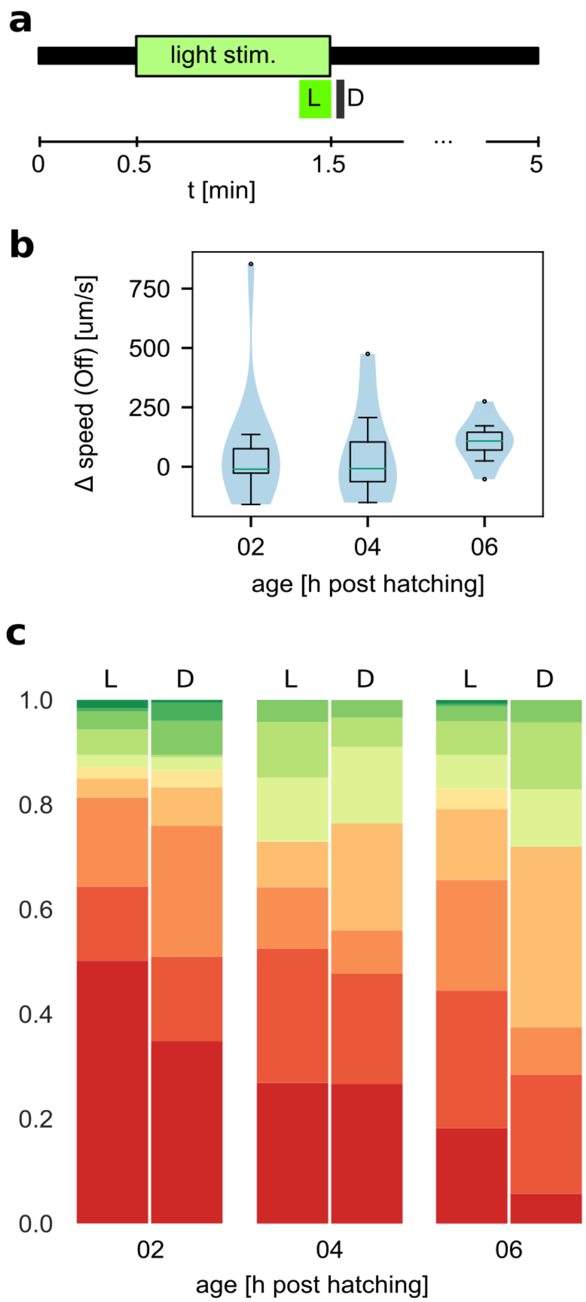

Supplemental Figure 5. Development of Light-Off response in animals reared and recorded at 14°C
(a) Experimental set-up. Light stimulus lasted 1 minute and the average speed of the animal in the last 10 s of the stimulation (L) was compared to the expected peak of speed upon onset of darkness (D) measured asspeed over 2.5 s starting from 0.5 s after light-off **(b)** Change in speed between L and D for different aged animals. **(c)** Distribution of behavioural modes in the last 10 s during a 1 min light stimulus – L and the onset of darkness – D for different aged animals (N_2=11, N_4=7, N_6=8)

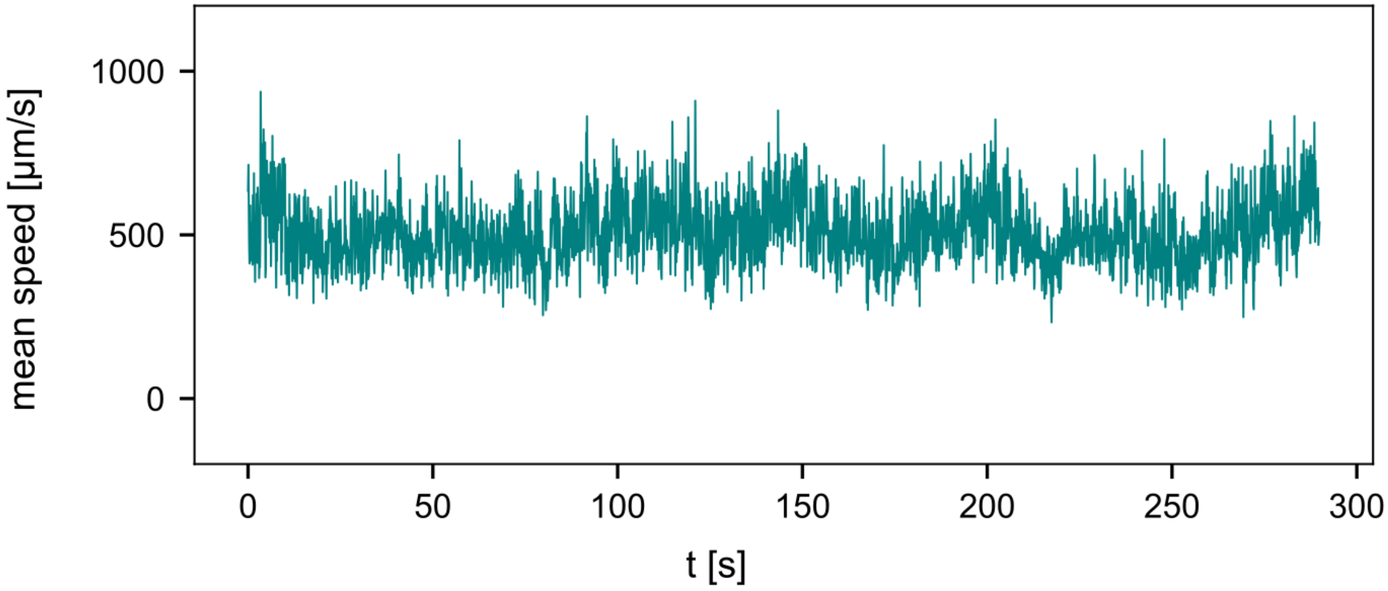

Supplemental Figure 6. Average speed in 5 minutes after acclimatization period
Average speed of 54 animals in the first 5 minutes after the acclimatisation period (recorded at 30frames/s and hence not directly comparable to the results presented for the 15 min acclimatisation period recorded at 10frames/s). Note that the average speed does not show further adaptation or other notable changes within the period.

a

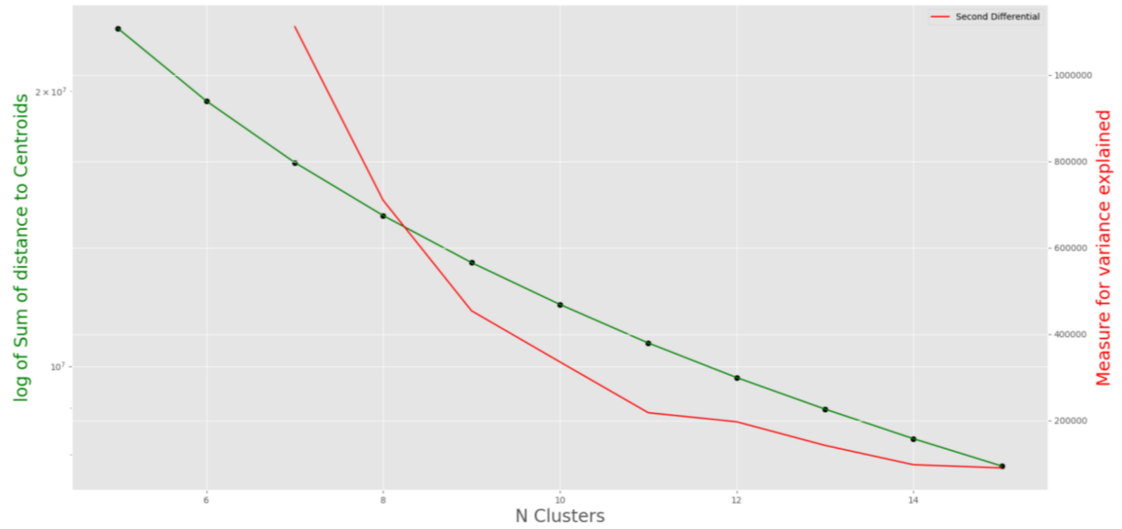

b

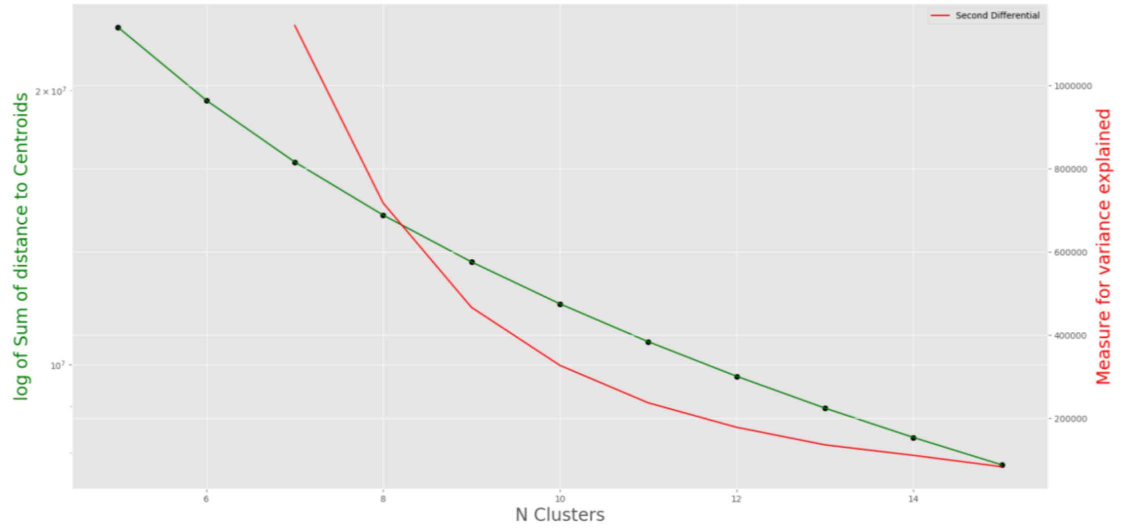

Supplemental Figure 7. Clustering methods

Variance explained in the dataset as a function of number of clusters is presented as the logarithm of the sum of distances to cluster centroids and its second differential. We present the data for the agglomerative clustering used in the paper **(a)** and k-means clustering **(b).**

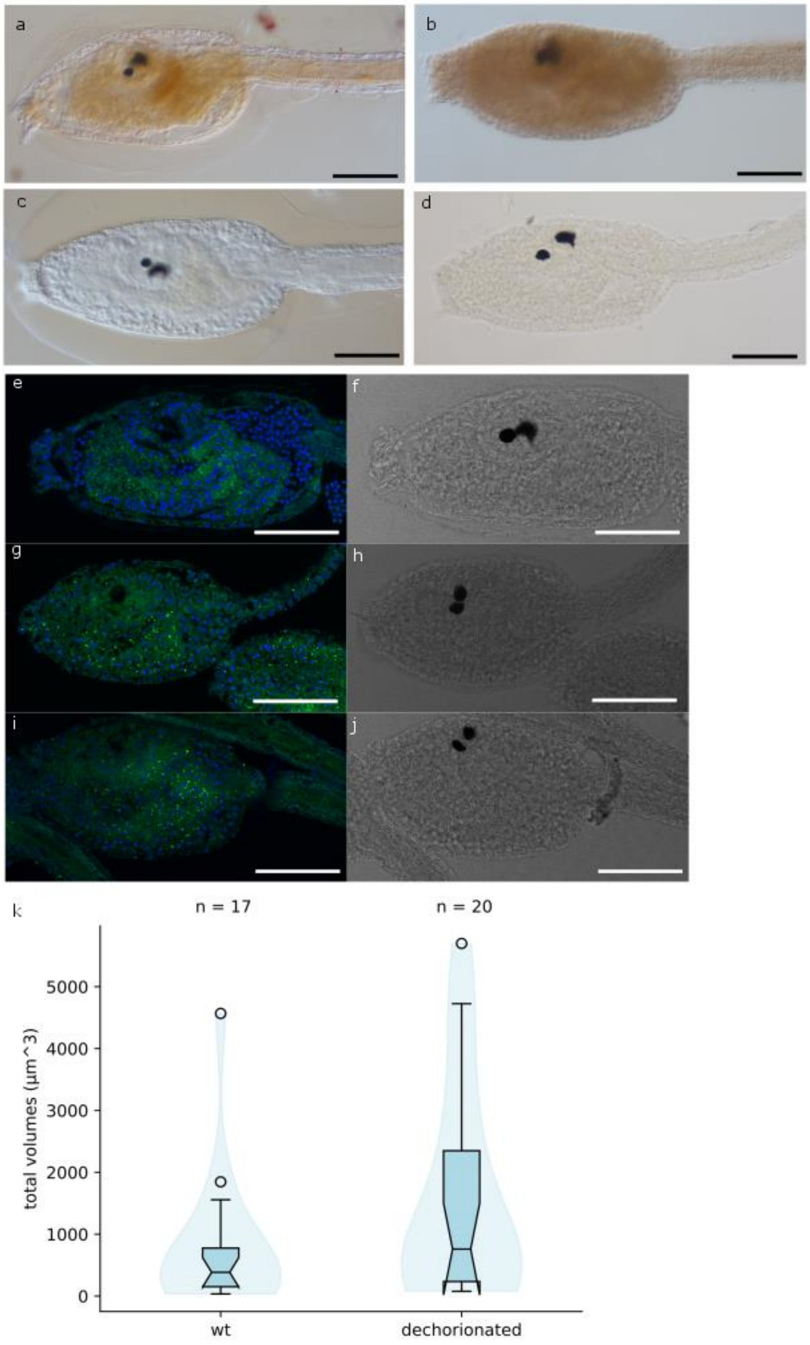

Supplemental Figure 8. Neutral Lipid analysis in chorionated and dechorionated animals
Dechorionated animals have a higher total volume of neutral lipids compared to chorionated animals. Representative Oil Red O stained **(a)** chorionated larva **(b)** dechorionated larva. Control (no Oil Red O stain) **(c)** chorionated larva, **(d)** dechorionated larva. BODIPY 493/503 staining was used to quantify neutral lipid stores. Representative confocal slices and transmitted light pictures of **(e,f)** chorionated larva **(g-j)** dechorionated larvae. Scale bars in panels (a-j) correspond to 50µm. **(k)** Quantification of total volume of BODIPY 493/503 signal from wt/chorionated (n=17) and dechorionated (n=20) larvae.

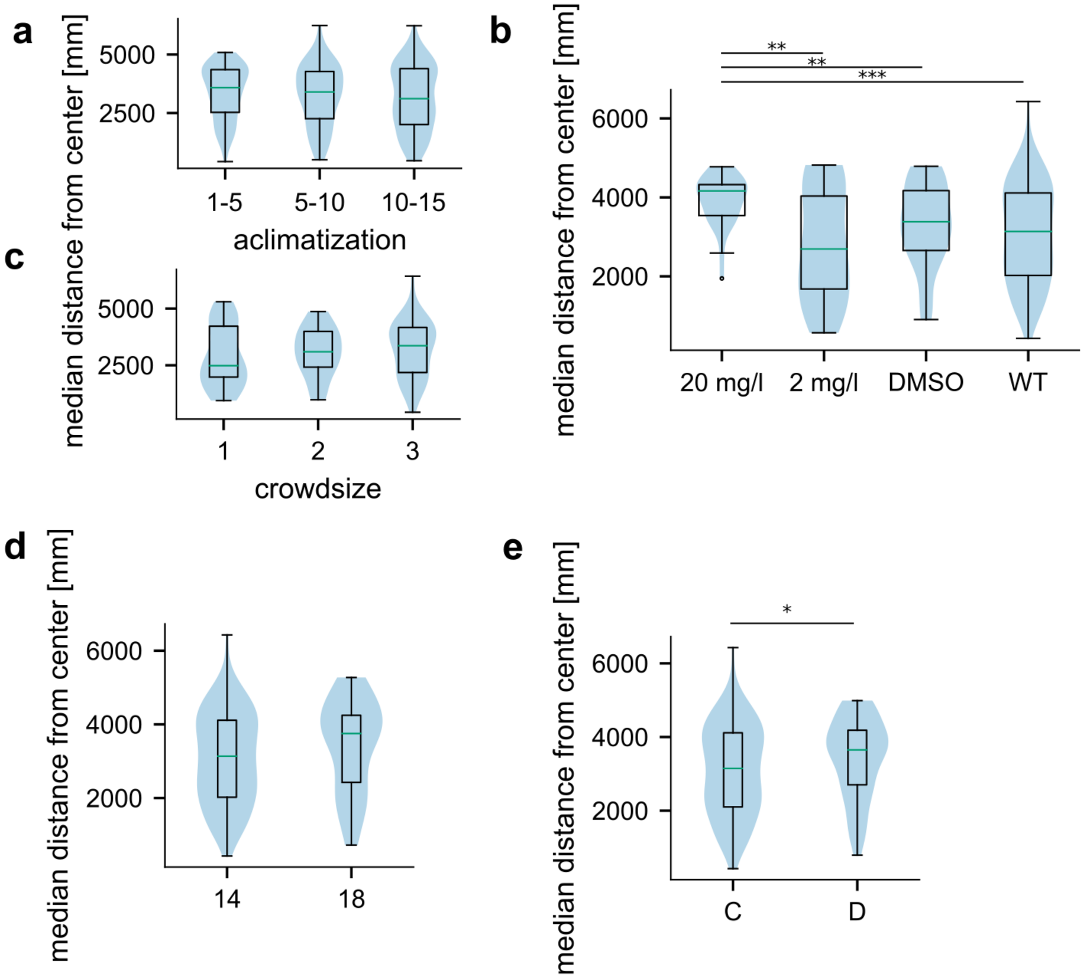

Supplemental Figure 9 Median distance from the arena centre for the different experiments:
As an additional measure of thigmotaxis, we used the median value for distance from the centre of the arena. The plots presented here use the same raw data as the main figures they refer to: **(a)** Acclimatization experiments (Fig. 2) (1-5 vs 5-10 *p*= 0.222259, 1-5 vs 10-15 *p*= 0.113126, 5-10 vs 10-15 *p*= 0.288749). **(b)** Modafinil experiments (Fig. 3) (20mg/l vs 2mg/l *p*= 0.002665, 20mg/l vs DMSO *p*= 0.004654, 20mg/l vs WT *p*= 0.000314). **(c)** Crowdsize experiment (1vs2 *p*= 0.265304, 1vs3 *p*= 0.168713, 2vs3 *p*=0.320831) (Fig. 4). **(d)** Temperature experiment (Fig. 6) *p*=0.068229, **(e)** dechorionation experiment (Fig. 7) *p*=0.022593. *p* values stated using Mann-Whitney U test.

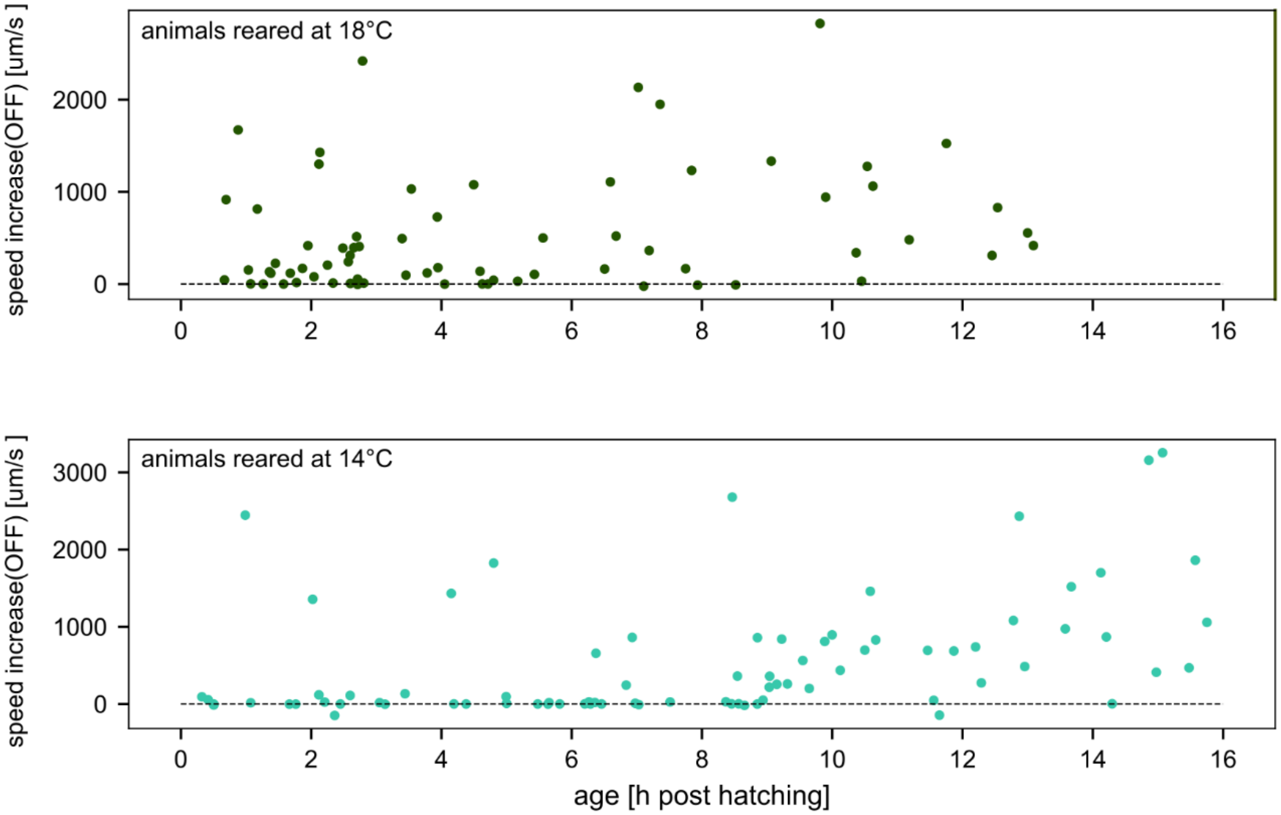

Supplemental Figure 10 Development of light- off response through age at 14°C and 18°C
Precisely staged animals were assayed for the development of a light-Off response. Larvae were reared either at 18°C (green, N=76 animals in 76 experiments, N(batches)=5) or at 14°C (light blue, N=83 animals in 83 experiments, N(batches)=5). The increase of speed after the end of a 1 min light stimulus is presented as the difference in median speed in the last 10s before light-OFF signal and 10 after light-OFF signal. Please note the rare occurrence of responses before age 6 h post fertilisation for animals at 14°C.

Supplemental Video 1

Behavioural mode 1

Examples video of an inactive animal. The coloured dots represent the animal's position during 50 frames based on which the current behavioural mode was calculated. In green are the 25 positions succeeding the current time-point and in red the 25 preceding it.

Supplemental Video 2

Behavioural modes 2 and 3

Examples video of an animal twitching, exhibiting behavioural modes 2 and 3. The coloured dots represent the animal's position during 50 frames based on which the current behavioural mode was calculated. In green are the 25 positions succeeding the current time-point and in red the 25 preceding it.

Supplemental Video 3

Behavioural modes 3, 4 and 8

Example video of an animal twitching, exhibiting behavioural modes 3, 4 and 8. The coloured dots represent the animal's position during 50 frames based on which the current behavioural mode was calculated. In green are the 25 positions succeeding the current time-point and in red the 25 preceding it.

Supplemental Video 4

Behavioural modes 1, 3, 4, 7, 8 and 9

Example video of an animal performing several different behavioural modes including the behavioural modes 7 and 9. The coloured dots represent the animal's position during 50 frames based on which the current behavioural mode was calculated. In green are the 25 positions succeeding the current time-point and in red the 25 preceding it.

Supplemental Video 5

Behavioural modes 5, 7, 9, 10 and 11

Example video of an animal performing several different behavioural modes. The coloured dots represent the animal's position during 50 frames based on which the current behavioural mode was calculated. In green are the 25 positions succeeding the current time-point and in red the 25 preceding it.
